# Reassessing alcohol consumption and cardiovascular disease by addressing bias in observational data: results from the multi-ethnic study of atherosclerosis

**DOI:** 10.1093/eurjpc/zwag201

**Published:** 2026-10-28

**Authors:** Shantanu Srivatsa, Elizabeth K. Farkouh, Tim Stockwell, James R. Pike, James M. Clay, Ganga S. Bey, Khurram Nasir, Matthew Budoff, Timothy Naimi, Michael E. Farkouh

**Affiliations:** 1 UNC Chapel Hill School of Medicine, University of North Carolina at Chapel Hill, 321 S Columbia St., Chapel Hill, NC 27599, USA;; 2 Department of Epidemiology, University of North Carolina at Chapel Hill, 135 Dauer Drive, Chapel Hill, NC 27599, USA;; 3 Mayo Clinic Alix School of Medicine, Mayo Clinic, 200 First St. SW, Rochester, MN, USA55905;; 4 Canadian Institute for Substance Use Research, University of Victoria, 2300 McKenzie Ave, Victoria, BC V8N 5M8, Canada;; 5 DeBakey Heart and Vascular Center, Houston Methodist, 6565 Fannin St, Houston, TX 77030, USA;; 6 Department of Medicine, Lundquist Institute at Harbor—UCLA,1124 W Carson St, Torrance, CA 90502, USA; and; 7 Academic Affairs, Cedars-Sinai Health System, 8700 Beverly Blvd, Los Angeles, CA 90048, USA

**Keywords:** Epidemiology, Alcohol, Atherosclerosis, Myocardial infarction, Stroke, Heart failure

## Abstract

**Aims:**

Alcohol and its relationship with cardiovascular disease (CVD) remain contentious. Several observational studies have found cardioprotective associations, while Mendelian randomization studies have found null or harmful effects of moderate drinking. Biases, including abstainer misclassification and sick-quitter effects, may obscure true associations. It is widely understood that former drinkers are often sick quitters. However, removal of former drinkers from analyses addresses bias in the abstainer group but fails to account for induced survivorship among current drinkers. This study sought to determine whether adjusting for biases altered the association between alcohol and CVD by comparing conventional models with those using (i) occasional drinkers as the reference group instead of lifetime abstainers and (ii) ‘intention-to-treat’ reallocation of former drinkers based on past drinking patterns.

**Methods and results:**

The Multi-Ethnic Study of Atherosclerosis is a prospective cohort study of 6814 participants aged 45–84 years between 2000 and 2002 without baseline CVD. A total of 6755 participants were included after excluding those with missing data. Alcohol consumption was self-reported and classified as lifetime abstainer, former, or current drinker [occasional (≤1 drink/week), light (2–7 drinks/week), moderate (8–14 drinks/week), heavy (>14 drinks/week)]. Outcomes included coronary artery calcium (CAC; 0, 1–100, 101–300, and >300) and clinical events [myocardial infarction (MI), stroke, cardiovascular mortality, composite major adverse cardiovascular events (MACEs), and heart failure]. Of 6755 participants, 20.6% were lifetime abstainers, 24.1% former drinkers, and 55.3% current drinkers. Adjusting for biases altered the observed relationship between alcohol and CVD, showing increased harm with light drinking, decreased protection with moderate drinking, and a dose–response relationship between alcohol and CAC, particularly CAC >300 in adjusted models. Protective associations between moderate drinking and MI [hazard ratio (HR): 0.64, 95% confidence interval (CI): 0.38–1.06] and cardiovascular mortality (HR: 0.81, 95% CI: 0.53–1.25) were attenuated and became non-significant. Light drinking shifted from null to increased risk for MI (HR: 1.46, 95% CI: 1.11–1.92), stroke (HR: 1.54, 95% CI: 1.17–2.03), MACE (HR: 1.33, 95% CI: 1.12–1.57), and heart failure (HR: 1.42, 95% CI: 1.10–1.84).

**Conclusion:**

Associations between alcohol and CVD in observational studies are sensitive to methodologic variation. Results from models correcting for abstainer/sick-quitter biases may help explain discrepant results from previous studies.

**Lay summary:**

The observed association between alcohol and cardiovascular disease is highly dependent on researchers’ choice of reference group and handling of past alcohol consumption.

- We utilized two main methodologic changes: (i) changing the reference group from abstainer (0 drinks/week) to occasional (1 drink/week) and (ii) placing the former drinker group into the current drinking group which best matches their consumption before they stopped drinking.
- Implementing these changes resulted in increased harm with light drinking, decreased protection with moderate drinking, and a dose–response relationship between alcohol and coronary artery calcium.

## Graphical Abstract

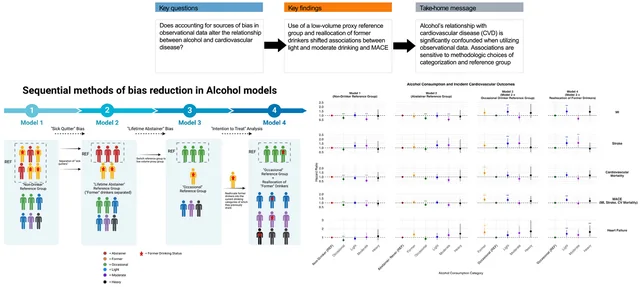

## Introduction

Alcohol use is a leading preventable cause of death and disability, but its association with cardiovascular disease (CVD) remains disputed.^1–3^ Observational studies comparing non-drinkers to low-volume drinkers generally find a J-shape relationship whereby consumption of up to one to two standard drinks (‘drinks’) per day confers protection against adverse outcomes.^4–7^ In contrast, other observational studies and Mendelian randomization studies have either found no association or a linear relationship between alcohol consumption and CVD risk.^8–10^

These discrepant results may be explained by confounding and misclassification in observational studies due to utilization of non-drinkers as a reference group. Compared with moderate drinkers, non-drinkers have higher prevalence of cardiovascular risk factors, including lower socioeconomic status and higher rates of diabetes and hypertension.^11,12^ Additionally, a significant number of non-drinkers are actually former drinkers who may have stopped drinking alcohol due to poor or worsening health, referred to as abstainer bias/sick-quitter effects.^12–14^ A 2024 study found that over 70% of systematic reviews or meta-analyses examining alcohol use and all-cause mortality were susceptible to sick-quitter bias by including former drinkers in the reference group.^15^

Scholars have often attempted to control for sick-quitter effects by separating former drinkers and utilizing a reference group of those who report never having consumed alcohol (‘lifetime abstainers’).^16–19^ However, this approach has significant limitations.

First, lifetime abstainers differ from drinkers across many factors, including socioeconomic status, religion, ethnicity, and immigration status.^20^ Furthermore, studies suggest that between 33 and 67% of those identifying as lifetime abstainers are actually former drinkers.^16,21–25^ In one study, 2% of lifetime abstainers had a documented alcohol-attributable condition, calling into question the validity of self-reported consumption in this group.^26^

In addition to concerns with lifetime abstainers, models separating former drinkers from the primary analysis generate an artificial cohort of healthy survivors who were able to sustain higher levels of alcohol consumption through the end of follow-up. Since outcomes from former drinkers should, in theory, accrue to drinking groups based on intention-to-treat (ITT) principles, systematically separating former drinkers removes participants who may have accumulated harm from alcohol use.^14,27^ This is similar to a per protocol approach and may potentially bias risk estimates away from the null, as former drinkers have accumulated risk and should be classified based on their former drinking pattern, not their current abstention.^28^ Thus, regardless of the validity of the ‘zero alcohol consumption’ in a lifetime abstainer group, removal of former drinkers from analyses after alcohol cessation only reduces bias in the lifetime abstainer group but fails to address survivorship bias among current drinkers.

Two important yet underutilized approaches to address these biases have been described. The first is to replace the lifetime abstainer reference group with a proxy abstainer group of very low-volume drinkers such as occasional drinkers (i.e. up to 1 drink per week).^14,29–33^ Occasional drinkers are unlikely to derive substantial biological effects at such low consumption levels and are more similar to regular drinkers than abstainers sociodemographically.^29^ The second approach involves reallocating former drinkers to their original drinking status by using an ‘ITT’ approach.^28,34^

The few studies which utilized either approach found differing results from the traditional J-shape curve, demonstrating attenuated protective associations, no effects, or non-significantly increased risk among moderate drinkers vs. lifetime abstainers.^34–36^ While a small number of studies have either utilized an occasional drinker reference group or reallocated former drinkers, to our knowledge, no study has examined alcohol and CVD associations using both methodologies.

This study assessed the relationship between alcohol consumption, coronary artery calcium (CAC), major adverse cardiovascular events (MACEs), and heart failure (HF) within the Multi-Ethnic Study of Atherosclerosis (MESA). Results were compared using traditional models employed in alcohol epidemiology vs. a novel methodology that accounted for various biases by (i) using very low-volume drinkers as a reference group and (ii) reallocating former drinkers back into their former consumption category.

## Methods

The MESA study design and methods have been previously described.^37^ The MESA is a multi-site prospective cohort study examining subclinical CVD in a diverse set of 6814 participants aged 45–84 years without known CVD at enrolment (2000–2002), recruited across six study centres. Our study population consisted of 6755 participants after excluding those with missing baseline alcohol use data. For analyses examining incident outcomes, between 6148 and 6175 participants were analysed after excluding participants with missing covariate/event data.

### Alcohol consumption

Baseline alcohol consumption was assessed using standardized questionnaires. Participants were categorized into lifetime abstainers, former drinkers, or current drinkers. We classified current drinkers into the following categories based on self-reported consumption: occasional (≤1 drink/week), light (2–7 drinks/week), moderate (8–14 drinks/week), and heavy (>14 drinks/week). Former drinkers reported on historical drinking patterns, allowing for reallocation into corresponding drinking categories (e.g. individual who formerly drank 6 drinks/week was reallocated as a light drinker).

### Models

**Figure 1:**
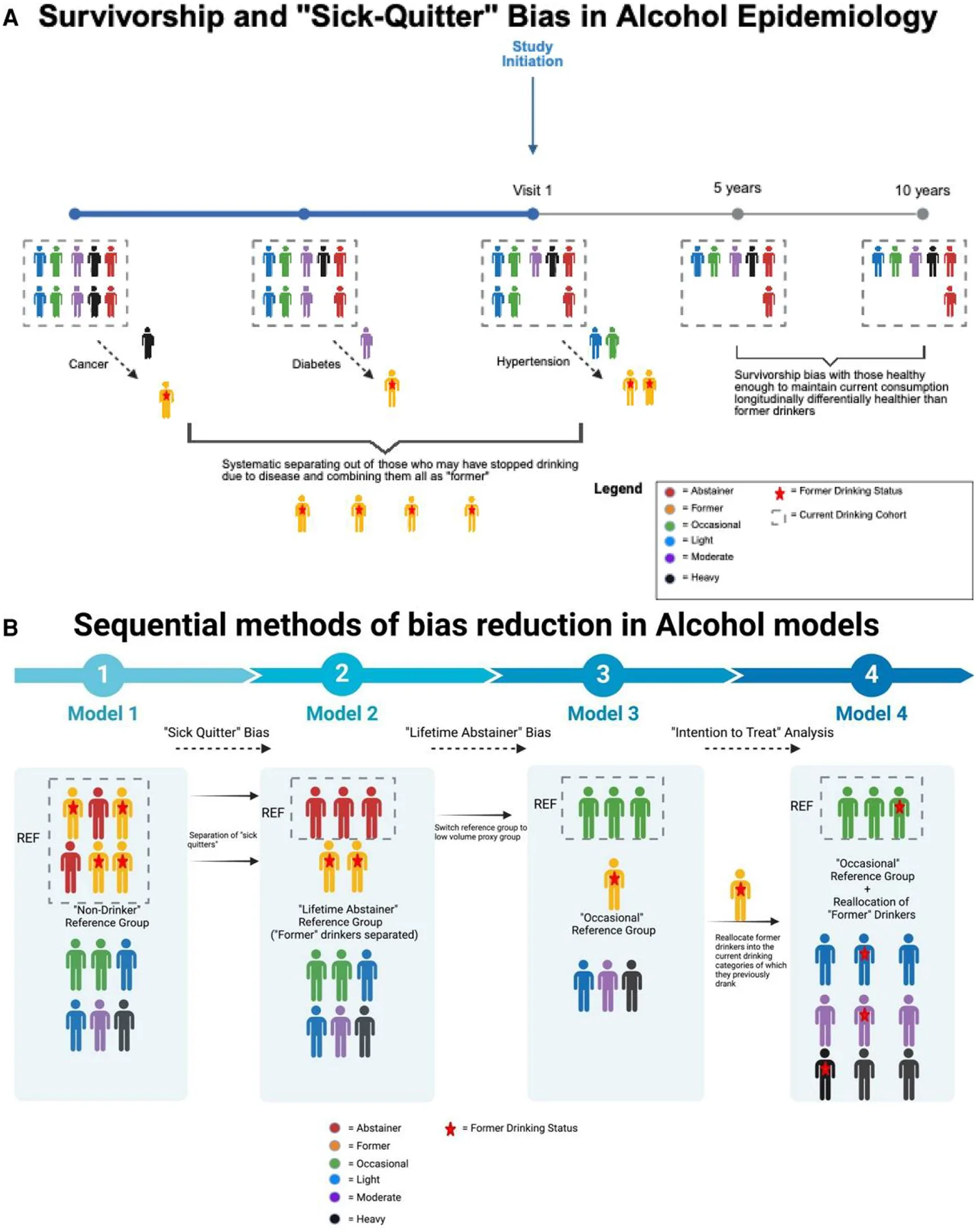
Theoretical model of sequential bias reduction. (*A*) Sick quitter bias. (*B*) Methods of bias reduction.

We compared four models that sequentially address potential biases found in self-reported, observational studies of alcohol and cardiovascular health. Model 1 represents an uncorrected model with the most bias, and Model 4 represents a model which has corrected for biases described previously. Model 1 utilized a ‘non-drinker’ reference group consisting of individuals reporting no current alcohol consumption. Model 2 partially addressed the ‘sick-quitter’ effect by separating lifetime abstainers from former drinkers, using lifetime abstainers as the reference group (traditional approach). Model 3 addressed the confounded/unreliable lifetime abstainer group (lifetime abstainer bias) by switching the choice of reference group from lifetime abstainer to occasional drinkers. Finally, Model 4 used Model 3’s reference group and reallocated former drinkers based on previous consumption. Figure 1 visually depicts these models.

### Covariates

Demographic variables included age, gender, race/ethnicity, education, income, health insurance, pack-years of smoking, medication use (blood pressure and lipid lowering), and history of disease (liver disease, cancer, and diabetes). Clinical variables included body mass index (BMI), systolic blood pressure (SBP)/diastolic blood pressure, LDL cholesterol, C-reactive protein (CRP), and fibrinogen. All covariates were selected from an *a priori* list of variables thought to be causally related to alcohol exposure and subsequently refined after likelihood ratio testing between nested models. Prior to model fitting, distributions of continuous covariates (including age, BMI, SBP, LDL, CRP, and fibrinogen) were examined for normality. Continuous variables were modelled as linear terms after confirming approximate linearity in the log-hazard or log-odds using residual plots; CRP was log-transformed prior to model inclusion. Proportional hazards assumptions for Cox models were assessed using visual inspection of log-minus-log survival plots. Influential observations were evaluated using deviance. Multicollinearity was assessed using variance inflation factor, with no covariates exceeding 10.

### Coronary artery calcium

The MESA computed tomography (CT) protocols are published elsewhere.^6^ An electron-beam CT scanner or a multi-detector CT system was used to assess CAC and scores were calculated using the Agatston Method. Coronary artery calcium scoring data between baseline examination and Examination 5 (2010–2012) were included. Cut-offs were chosen based on previous studies which found comparable event rates between individuals with CAC scores >300 and those with established atherosclerotic cardiovascular disease.^38,39^ Scores were categorized into ‘none’ (0 Agatston units), ‘mild’ (1–100), ‘moderate’ (101–300), and ‘severe’ (>300) plaque.

### Cardiovascular events

Trained MESA personnel abstracted medical records for potential cardiovascular events which were subsequently adjudicated by physician review. Detailed MESA event methodology is described elsewhere.^40^ For this study, we examined MACEs [composite of myocardial infarction (MI), stroke, and cardiovascular death] and HF. Individual components of the MACE outcome were also investigated independently.

### Statistical analysis

Baseline characteristics are presented descriptively across alcohol consumption categories without formal hypothesis testing. Associations between alcohol consumption and (i) baseline CAC categories and (ii) incident cardiovascular events, including composite MACE, its individual components (MI, stroke, and cardiovascular death), and HF were analysed for four models progressively addressing bias (Figure 1). We used multinomial logistic regression for CAC scores and time-to-event survival models for incident events, adjusting for all covariates except health insurance. Since ascertainment of HF events required both a physician diagnosis as well as the patient being on treatment, we additionally adjusted for health insurance for HF. Health insurance was found to be a significant covariate for incident HF models but not for any other subclinical/clinical outcome.

Time-to-event analysis was conducted using cause-specific Cox proportional hazards models and Fine–Gray competing risk models. Survival curves were generated using Kaplan–Meier estimators for cause-specific models with log-rank tests with Bonferroni corrections for comparisons between individual survival curves.

#### Sensitivity and stratified analyses

Sensitivity analyses included multinomial logistic regression models using alternative CAC score cut-offs (0, 1–10, 11–99, and ≥100). Stratified analyses, by gender adjusting for covariates as described previously, were conducted. Longitudinal stability of the abstainer group was tested by excluding 187 abstainers who reported alcohol consumption at subsequent examination visits. All sensitivity analyses are shown in the Supplementary material.

#### Software

All analyses were conducted using RStudio (version 2024.12.0 +467), with the *nnet*, *survival*, and *cmprsk2* packages. Visualization was performed using *ggpubr*, *ggplot2*, and *ggforest* packages. A two-sided *P*-value <0.05 was considered statistically significant.

## Results

### Baseline characteristics

Among 6755 participants, 20.6% were lifetime abstainers, 24.1% were former drinkers, and 55.3% were current drinkers. Within current drinkers, 34.2% were occasional (≤1 drink/week), 25.5% were light (2–7 drinks/week), 7.9% were moderate (8–14 drinks/week), and 4.2% were heavy (>14 drinks/week) drinkers. Table 1 provides baseline characteristics across alcohol consumption categories.

Gender and race/ethnicity varied across alcohol consumption categories (Table 1). Among abstainers, 76.6% were female, compared with 57.8% of occasional drinkers and only 12.3% of heavy drinkers. Abstainers were more likely to be Chinese, former drinkers were more likely to be Black, and heavy drinkers were primarily White. Former drinkers had the highest prevalence of diabetes (17.4%), while heavy drinkers had the lowest (5.5%). Abstainers and former drinkers had higher systolic blood pressure and had lower income, education, and insurance rates compared with current drinkers.

### Coronary artery calcium outcomes

**Figure 2:**
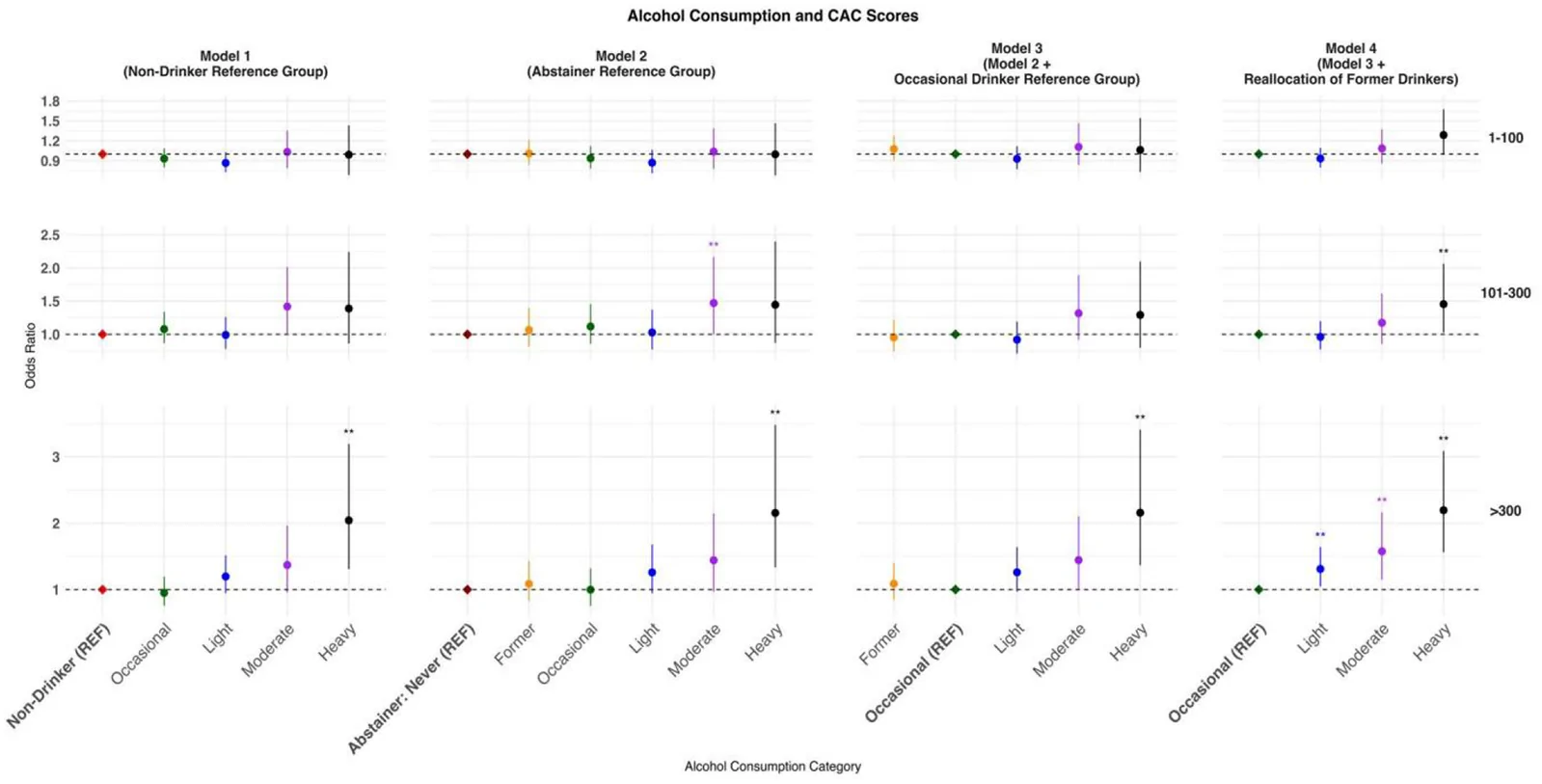
Forest plots displaying odds ratios for the association between drinking status and categorized coronary artery calcium (CAC) scores (1–100, 101–300, and >300 Agatston units). The figure compares an uncorrected traditional model (Model 1) against progressively bias-adjusted models (Models 2–4) using multinomial logistic regression.

Among the baseline categories of CAC, 50.6% of participants had no plaque, 26.5% had mild plaque (1–100), 11.2% had moderate plaque (101–300), and 11.7% had severe plaque (>300). Figure 2 and Table 2 show the associations between alcohol consumption and CAC scores from multinomial logistic regression models. Across four sequential models, heavy drinking was associated with similarly higher odds of severe plaque, when compared with no plaque. Model 4 was the only model in which light, moderate, and heavy drinking were significantly associated with higher odds of CAC (>300) compared with no CAC. For mild and moderate plaque, dose–response between alcohol and CAC burden became sequentially more prominent with model progression.

### Cardiovascular events

**Figure 3:**
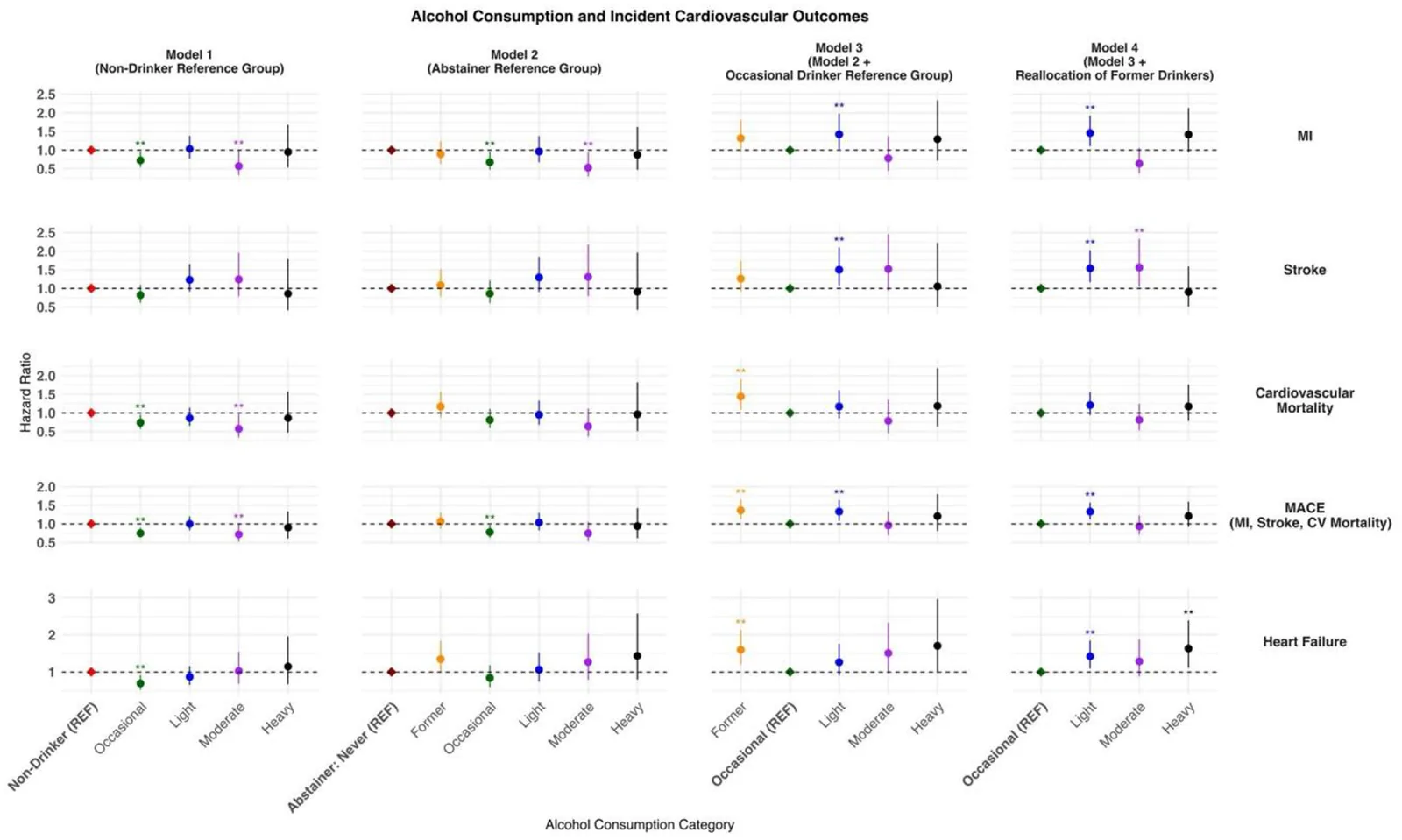
Alcohol consumption and incident cardiovascular outcomes.

**Figure 4:**
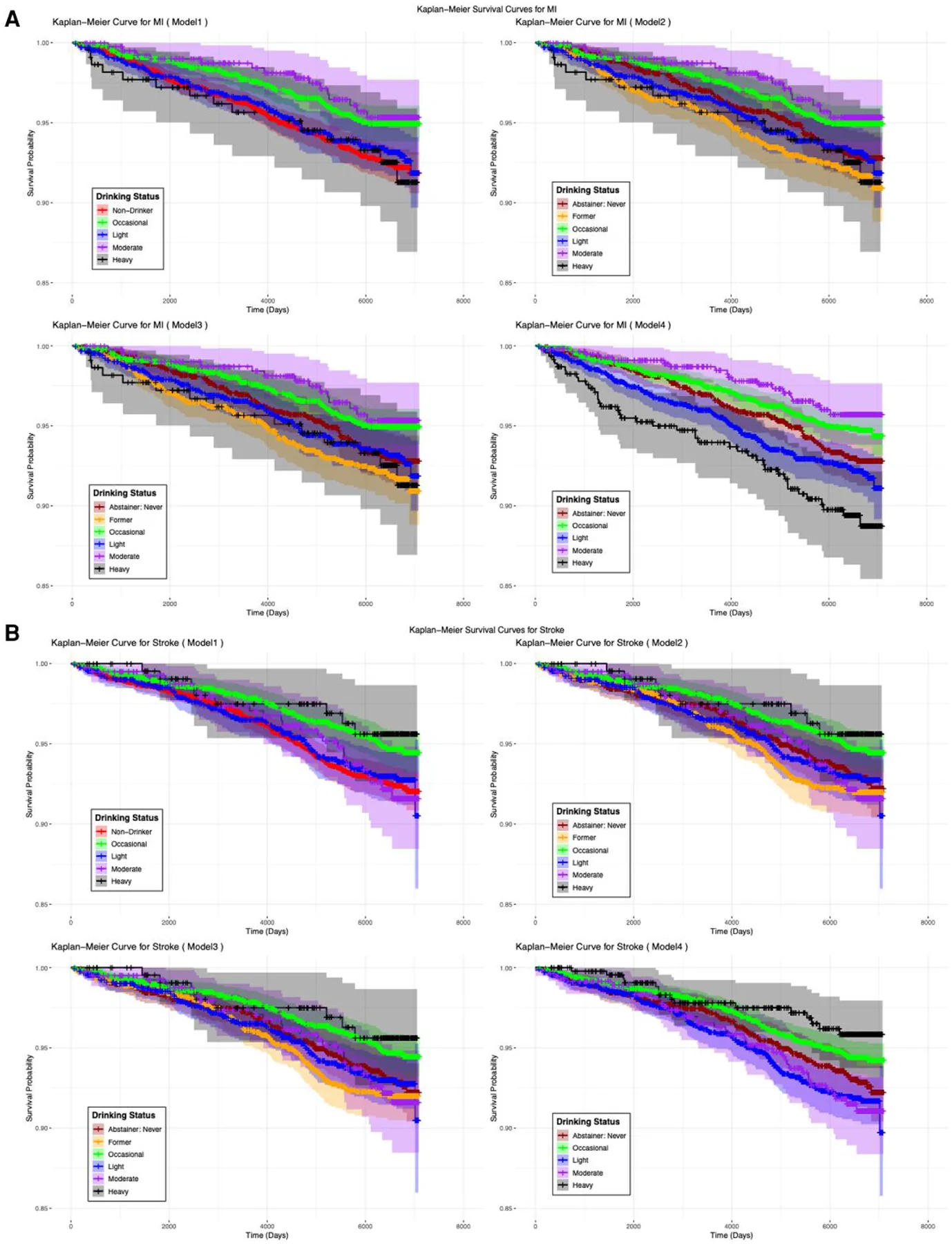

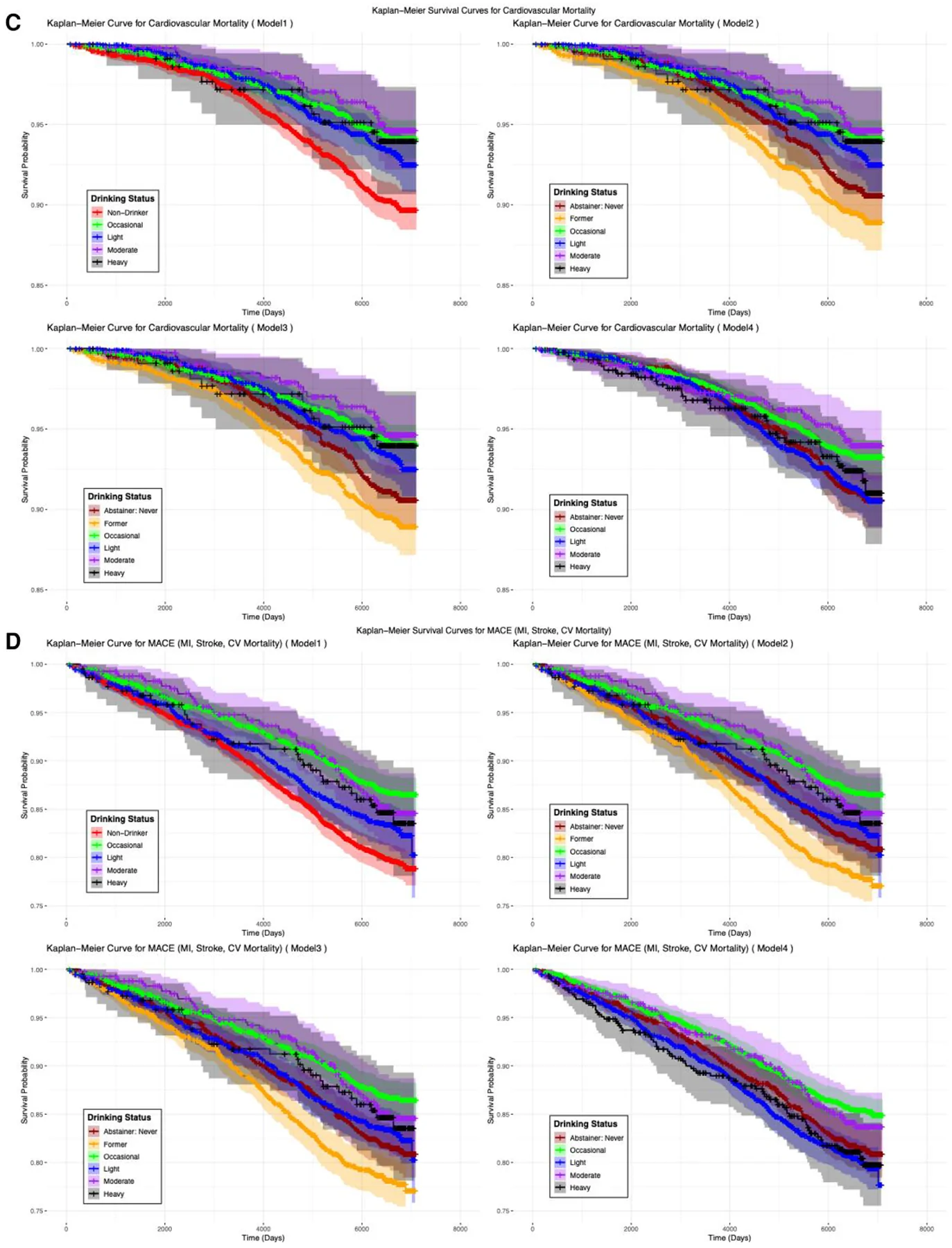

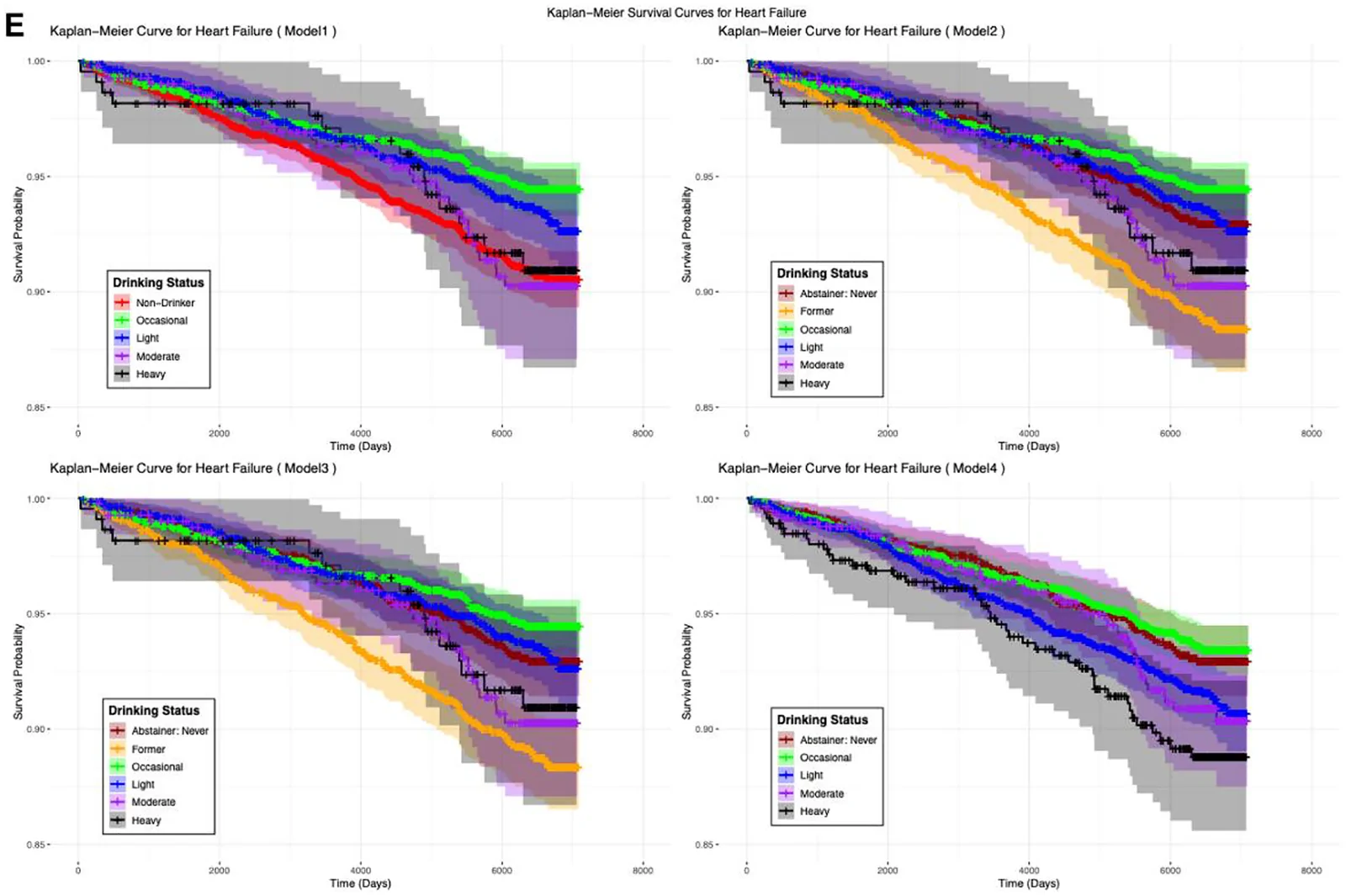
(*A–E*) Survival curves by alcohol consumption and incident cardiovascular outcomes.

Figure 3 shows cause-specific Cox regression models between alcohol consumption and incident outcomes, and Kaplan–Meier plots are visualized in Figure 4. Median follow-up time was 6459 days (interquartile range: 4534–6746; Table 3).

#### Myocardial infarction

There were 374 incident MIs throughout follow-up. In traditional models, moderate alcohol consumption was associated with lower risk of MI compared with non-drinkers/lifetime abstainers [Model 1: hazard ratio (HR): 0.57, 95% confidence interval (CI): 0.33–0.98; Model 2: HR: 0.53, 95% CI: 0.29–0.95]. In bias-attenuated models, however, these protective associations were weakened and lost statistical significance (Model 3: HR: 0.78, 95% CI: 0.44–1.37; Model 4: HR: 0.64, 95% CI: 0.38–1.06). Light drinking was associated with increased risk of MI in bias-attenuated models (Model 3: HR: 1.43, 95% CI: 1.02–1.98; Model 4: HR: 1.46, 95% CI: 1.11–1.92).

#### Stroke

There were 377 incident stroke events throughout follow-up. In traditional models, alcohol consumption was not significantly associated with stroke risk. However, in bias-attenuated models, light drinking (Model 3: HR: 1.50, 95% CI: 1.08–2.10; Model 4: HR: 1.54, 95% CI: 1.17–2.03) was associated with an increased risk of stroke. In Model 4, moderate drinking was associated with increased risk of stroke (HR: 1.56, 95% CI: 1.05–2.33). Heavy drinking was not significantly associated with stroke risk in any model.

#### Cardiovascular mortality

There were 457 deaths due to cardiovascular causes throughout follow-up. In Model 1, moderate drinking had a protective association (HR: 0.57, 95% CI: 0.34–0.97) with cardiovascular mortality, but this association was attenuated and non-significant in bias-adjusted models (Model 3: HR: 0.79, 95% CI: 0.46–1.35; Model 4: HR: 0.81, 95% CI: 0.53–1.25). Former drinking, when compared with occasional drinking, was associated with increased risk of cardiovascular mortality in Model 3 (HR: 1.44, 95% CI: 1.09–1.91).

#### Major cardiovascular events

There were 997 incident composite MACE (MI, stroke, and CV mortality) events throughout follow-up. Moderate drinking was associated with a lower risk of MACE in traditional models (Model 1: HR: 0.72, 95% CI: 0.63–0.90; Model 2: HR: 0.75, 95% CI: 0.53–1.06). However, this protective association was attenuated and non-significant in Models 3 (HR: 0.96, 95% CI: 0.69–1.33) and 4 (HR: 0.93, 95% CI: 0.71–1.23). In Model 4, light drinking was associated with an increased risk of MACE (HR: 1.33, 95% CI: 1.12–1.57).

#### Heart failure

There were 433 incident HF events throughout follow-up. Light and heavy drinking were not significantly associated with HF in Models 1 and 2. However, in Model 4 light (HR: 1.42, 95% CI: 1.10–1.84) and heavy drinking (HR: 1.63, 95% CI: 1.11–2.40) were associated with increased HF risk. Former drinkers, vs. occasional drinkers had significantly higher risk for HF (Model 3: HR: 1.60, 95% CI: 1.20–2.14).

#### Kaplan–Meier survival curves

Figure 4 demonstrates survival curves by model and outcome. As models progressed, survival curves tended to shift such that survival decreased with increasing alcohol consumption, with the exception of moderate drinking which had the highest survival for MI and cardiovascular mortality, and had longer time-to-event for MI when compared with light drinking (*P* = 0.04). Across all models, non-drinkers (Model 1) and former drinkers (Models 2/3) had significantly shorter time to event, most pronounced in cardiovascular mortality, MACE, and HF. Within stroke, light and moderate drinking had the lowest survival, and heavy drinking the greatest, although no subgroup differences were statistically significant.

### Sensitivity analyses

Sensitivity analyses using alternative CAC cut-offs (0, 1–10, 11–99, ≥100) and competing risk analysis using Fine–Gray subdistribution hazard modelling and cumulative incidence curves confirmed robustness of primary analyses (see Supplementary material).

## Discussion

In the present study, we investigated how associations between alcohol and CVD are affected by addressing potential biases encountered in observational studies. Our study demonstrates that shifting the reference group to occasional drinkers and reallocating former drinkers based on prior consumption attenuated protective associations and uncovered potential harms of low-volume drinking. Our results may help explain conflicting findings between observational and genetic experimental studies. Our results also highlight the fragility of observational studies and sensitivity of observed associations to study methodology.

The demographic characteristics, incident events, and survival curves in our study indicated that former drinkers and abstainers tended to have higher rates of comorbidities and disease, greater risk for incident cardiovascular events, and overall lower survival. This likely reflects survivorship bias, where individuals with poorer health are overrepresented in the non-drinker groups, while those who are able to continue light or moderate drinking are healthier from the onset.^34^ These data provide support to the methodological adjustment for ‘sick quitter’/‘abstainer’ biases.

Within CAC outcomes, a dose–response relationship was found at the >300 CAC level regardless of model. Notably, the bias-adjusted models helped uncover a dose–response pattern at mild and moderate plaque.

Our findings raise more skepticism regarding the existence of a J-shaped association between alcohol and CVD. ‘Light drinking’ may be interpreted by the general public as ‘safe’ or ‘below risk.’ However, in our fully adjusted models light drinking was associated with an increased risk of MI, stroke, MACE, and HF. On the other hand, despite the dose–response relationship with CAC, moderate drinking did not show consistent harm or benefit for MI or cardiovascular mortality. This divergence may reflect alcohol’s theorized plaque stabilization, anti-platelet, or vasodilatory effects at doses where cardiovascular harms and physiologic benefits for these outcomes balance one another. However, threshold effects did not occur uniformly across outcomes. While moderate drinking showed an attenuated/null association for MI and cardiovascular mortality, it was associated with a 57% increased risk of stroke in fully adjusted models.

Interestingly, although alcohol use exhibited a strong dose–response relationship with CAC data at baseline, there was not a strong dose–response relationship with regards to events, particularly for heavy use. The observed dose–response relationship between alcohol consumption and CAC, particularly for CAC >300, likely represents cumulative vascular injury which is known to be a strong prognostic indicator for risk of acute events. The observed divergence between CAC findings and incident outcomes with respect to alcohol consumption may reflect competing biological effects of alcohol on thrombosis, vascular tone, and plaque stability that differentially influence subclinical atherosclerosis vs. clinical presentation. In addition, smaller sample size and longitudinal instability of the heavy drinking group may have contributed to the lower risk observed in the heavy consumption category, as a majority of the 220 heavy drinkers at baseline did not maintain their consumption levels over an average follow-up time of ~15 years. Studies have found that heavy drinkers are extremely unstable longitudinally, and light/moderate drinkers are more likely to maintain consistent consumption over time.^41^

From a clinical and public health perspective, these results underscore that both protective and harmful associations in observational studies may be highly contingent on analytic choices. Our findings should not be interpreted as prescriptive guidance for individual patients, but rather as evidence that the use of observational data to inform counselling and guidelines warrant careful scrutiny and methodological sensitivity analyses. Where possible, randomized data should be prioritized, such as the recent randomized trial examining alcohol consumption and atrial fibrillation.^42^

Future observational studies examining alcohol consumption at a minimum should utilize sensitivity analyses incorporating a low-volume proxy reference group and ‘ITT’ reallocation, avoiding sole use of non-drinker or lifetime abstainer reference groups when possible.

### Strengths and limitations

Strengths of our study include a large, racially/ethnically diverse cohort with comprehensive follow-up on subclinical and clinical disease. There are several limitations of our study. First, as with any associations derived from observational data, we do not assert causality for any findings presented. Our results challenge the paradigm of the ‘J-shape’ but do not disprove cardioprotective effects of light-to-moderate alcohol intake. Due to the small number of incident events within each group after stratifying by different alcohol consumption levels, replication of our results in other large cohorts, improved survey methodology, and novel measures of alcohol consumption are needed to understand the impacts of alcohol on CVD. Randomized evidence, scarce in alcohol epidemiology, is critically needed to truly elucidate impacts of alcohol use.^42^

Although MESA is racially and ethnically diverse, drinking behaviours, social norms, and patterns of alcohol use vary substantially across cultural and geographic contexts. These findings should therefore be interpreted with caution in populations with different drinking distributions, beverage preferences, or social determinants of alcohol use. The methodological sensitivity demonstrated here is nonetheless likely applicable and should be tested across settings, as abstainer misclassification and survivorship bias are common features of observational alcohol research.

Although our study accounts for measures of lifetime consumption, there remains residual measurement bias through dichotomization of drinkers into ‘former’ and ‘current.’ Our reference group of occasional drinkers may still suffer from residual bias due to uncollected former drinking history (e.g. may have drunk heavily in the past). Furthermore, there is likely differential measurement error by ‘former’ vs. ‘current’ drinking status, in which for a particular level of consumption, former drinkers may be more likely to underreport or recall past consumption with less accuracy than those with current drinking consumption. However, retaining former drinkers as abstainers or excluding them entirely also imposes strong assumptions and may exacerbate survivorship bias by selectively removing individuals who accumulated alcohol-related risk prior to cessation. Examining Model 3, we note that point estimates shift with the sole implementation of the reference group change, thus providing reassurance that shifts in estimates are not entirely dependent upon reclassification of former drinkers’ past consumption.

Alcohol consumption is known to change over time, particularly among heavier drinkers. Although longitudinal modelling of time-varying exposure is desirable, we opted to restrict analyses to baseline exposure in order to demonstrate the shifting of point estimates with our methodology in relation with previously conducted observational studies which nearly universally rely upon baseline exposure data. Thus, our estimates in Models 3 and 4 are likely still biased due to lack of addressing time-varying confounding which may lead to reduction or increase in alcohol intake longitudinally. In addition, we did not incorporate longitudinal stability of alcohol consumption. Studies have found that in particular, heavy drinking is less stable than abstinence or moderate drinking in studies with longer follow-up.^41^ Limiting analyses to a small subset who maintained the same consumption levels would induce survivorship bias. Nevertheless, reallocation does account for changes/discontinuation in consumption that may occur longitudinally.

We also did not examine associations by alcoholic beverage type (wine, beer, etc.), as we primarily sought to understand the impact of ethanol on CVD. As ethanol is the common component among beverage types, benefits between alcohol sub-types should be attributable to non-ethanol components of these beverages.

## Conclusions

These findings contribute to growing evidence suggesting that traditional observational studies are subject to measurement error, and associations between alcohol consumption and cardiovascular outcomes in observational studies are sensitive to variations in methodology. In our study, adjusting for some of these biases attenuated some cardioprotective associations while also uncovering potential harm at low alcohol consumption levels. Future observational studies should seek to utilize similar methodology with occasional drinkers as a reference group and reallocation of former drinkers according to previous drinking status.

## Supplementary Material

supplementary data

Supplementary material is available at European Journal of Preventive Cardiology.

## Figures and Tables

**Table 1:** Baseline characteristics of 6755 MESA study participants stratified by self-reported drinking status

	Abstainer (*n* = 1390)	Former (*n* = 1624)	Current occasional (≤1 drink/week) (*n* = 1781)	Current light (1–7 drinks/week) (*n* = 1329)	Current moderate (8–14 drinks/week) (*n* = 411)	Current heavy (>14 drinks/week) (*n* = 220)
**Age at enrolment, mean (SD)**	63.9 ± 10.2	62.9 ± 10.2	60.8 ± 10.2	61.7 ± 10.2	62.6 ± 10.1	59.4 ± 9.6
**Sex, *n* (%)**						
** Female**	1065 (76.6)	764 (47.0)	1029 (57.8)	556 (41.8)	206 (34.5)	27 (12.3)
** Male**	325 (23.4)	860 (53.0)	752 (42.2)	773 (58.2)	391 (65.5)	193 (87.7)
**Race/ethnicity, *n* (%)**						
** White**	246 (17.7)	484 (29.8)	750 (42.1)	707 (53.2)	357 (59.8)	148 (67.3)
** Chinese**	432 (31.1)	118 (7.3)	177 (9.9)	59 (4.4)	22 (3.7)	5 (2.3)
** Black**	324 (23.3)	620 (38.2)	481 (27.0)	332 (25.0)	126 (21.1)	30 (13.6)
** Hispanic/Latino**	388 (27.9)	402 (24.8)	373 (20.9)	231 (17.4)	92 (15.4)	37 (16.8)
**Education, *n* (%)**						
** ≥Bachelor’s degree**	321 (23.1)	438 (27.0)	706 (39.6)	634 (47.7)	193 (47.0)	92 (41.8)
**Income, *n* (%)**						
** ≥$75 000**	128 (9.6)	215 (14.0)	429 (24.8)	460 (35.5)	158 (39.3)	79 (36.4)
**Insurance, *n* (%)**						
** Uninsured**	204 (14.7)	144 (8.9)	134 (7.5)	79 (5.9)	20 (4.9)	15 (6.8)
**Body mass index kg/m** ^**2**^ **, mean (SD)**	27.9 ± 5.7	29.2 ± 5.8	28.6 ± 5.6	27.7 ± 4.9	27.5 ± 4.8	28.1 ± 4.3
**Seated systolic blood pressure (mmHg), mean (SD)**	129.6 ± 22.9	128.1 ± 21.8	124.4 ± 20.6	124.9 ± 20.8	125.2 ± 21.2	125.7 ± 17.9
**Seated diastolic blood pressure (mmHg), mean (SD)**	70.9 ± 10.3	72.1 ± 10.4	71.2 ± 9.9	72.5 ± 10.3	73.6 ± 10.4	76.1 ± 9.7
**Blood pressure medication use, *n* (%)**	519 (37.3)	630 (38.8)	535 (30.0)	364 (27.4)	129 (31.4)	64 (29.1)
**Diabetes, *n* (%)**	197 (14.2)	283 (17.4)	165 (9.3)	83 (6.2)	25 (6.1)	12 (5.5)
**Cancer, *n* (%)**	87 (6.3)	132 (8.1)	124 (7.0)	134 (10.1)	39 (9.5)	14 (6.4)
**Liver disease, *n* (%)**	51 (3.7)	77 (4.7)	44 (2.5)	40 (3.0)	16 (3.9)	4 (1.8)
**Smoking (pack-years), mean (SD)**	3.7 ± 13.2	4.3 ± 7.1	4.0 ± 5.9	3.2 ± 5.0	3.1 ± 4.3	3.3 ± 6.0
**Lipid-lowering medication use, *n* (%)**	223 (16.0)	265 (16.3)	298 (16.8)	220 (16.6)	63 (15.3)	29 (13.2)
**LDL cholesterol (mg/dL), mean (SD)**	118.0 ± 32.0	115.9 ± 31.4	118.1 ± 31.4	117.2 ± 30.6	115.8 ± 31.6	117.8 ± 31.7
**C-reactive protein (mg/L), mean (SD)**	3.7 ± 5.0	4.3 ± 7.1	4.0 ± 5.9	3.2 ± 5.0	3.1 ± 4.3	3.3 ± 6.0
**Fibrinogen (mg/dL), mean (SD)**	360.5 ± 71.7	357.4 ± 79.4	347.1 ± 71.9	332.1 ± 68.5	321.7 ± 69.3	317.6 ± 67.9

**Table 2:** Alcohol consumption and baseline CAC score outcomes

CAC outcome	Drinking level	OR (95% CI)
1–100		
Model 1 (non-drinker reference group)	Non-drinker (Ref.)	1.0 (Ref.)
	Occasional	0.93 (0.8–1.08)
	Light	0.87 (0.73–1.03)
	Moderate	1.03 (0.79–1.36)
	Heavy	0.99 (0.69–1.43)
Model 2 (abstainer reference group)	Abstainer: never (Ref.)	1.0 (Reference)
	Former	1.01 (0.84–1.21)
	Occasional	0.94 (0.78–1.12)
	Light	0.87 (0.71–1.06)
	Moderate	1.04 (0.78–1.39)
	Heavy	1 (0.68–1.46)
Model 3 (Model 2 + occasional drinker reference group)	Former	1.08 (0.91–1.28)
	Occasional (Ref.)	1.0 (Ref.)
	Light	0.93 (0.77–1.12)
	Moderate	1.11 (0.84–1.47)
	Heavy	1.06 (0.73–1.54)
Model 4 (Model 3 + reallocation of former drinkers)	Occasional (Ref.)	1.0 (Ref.)
	Light	0.93 (0.8–1.09)
	Moderate	1.09 (0.86–1.38)
	Heavy	1.29 (0.99–1.68)
101–300		
Model 1 (non-drinker reference group)	Non-drinker (Ref.)	1.0 (Ref.)
	Occasional	1.08 (0.87–1.34)
	Light	0.99 (0.78–1.26)
	Moderate	1.42 (1–2.02)
	Heavy	1.39 (0.86–2.24)
Model 2 (abstainer reference group)	Abstainer: never (Ref.)	1.0 (Ref.)
	Former	1.07 (0.81–1.39)
	Occasional	1.12 (0.86–1.46)
	Light	1.03 (0.77–1.37)
	**Moderate**	*1.47 (1.00–2.17)*
	Heavy	1.44 (0.87–2.4)
Model 3 (Model 2 + occasional drinker reference group)	Former	0.95 (0.75–1.22)
	Occasional (Ref.)	1.0 (Ref.)
	Light	0.92 (0.71–1.19)
	Moderate	1.32 (0.92–1.89)
	Heavy	1.29 (0.8–2.1)
Model 4 (Model 3 + reallocation of former drinkers)	Occasional (Ref.)	1.0 (Ref.)
	Light	0.96 (0.77–1.2)
	Moderate	1.17 (0.86–1.61)
	**Heavy**	*1.45 (1.03–2.06)*
>300		
Model 1 (non-drinker reference group)	Non-drinker (Ref.)	1.0 (Ref.)
	Occasional	0.95 (0.76–1.19)
	Light	1.2 (0.95–1.52)
	Moderate	1.37 (0.96–1.96)
	**Heavy**	*2.04 (1.31–3.19)*
Model 2 (abstainer reference group)	Abstainer: never (Ref.)	1.0 (Ref.)
	Former	1.09 (0.83–1.43)
	Occasional	1 (0.75–1.32)
	Light	1.26 (0.94–1.68)
	Moderate	1.44 (0.97–2.15)
	**Heavy**	*2.15 (1.33–3.48)*
Model 3 (Model 2 + occasional drinker reference group)	Former	1.09 (0.84–1.4)
	Occasional (Ref.)	1.0 (Ref.)
	Light	1.26 (0.97–1.64)
	Moderate	1.44 (0.99–2.1)
	**Heavy**	*2.16 (1.37–3.41)*
Model 4 (Model 3 + reallocation of former drinkers)	Occasional (Ref.)	1.0 (Ref.)
	**Light**	*1.31 (1.05–1.64)*
	**Moderate**	*1.58 (1.15–2.16)*
	**Heavy**	*2.2 (1.56–3.09)*

Odds ratios for the association between drinking status and categorized coronary artery calcium (CAC) scores (1–100, 101–300, and >300 Agatston units). The models begin with an uncorrected traditional model (Model 1) and progressively address abstainer and sick-quitter biases (Models 2–4) using multinomial logistic regression. Bolded cells refer to statistically significant associations at a two-sided α = 0.05 threshold.

**Table 3:** Alcohol consumption and incident cardiovascular outcomes

Outcome and model	Alcohol consumption category	HR (95% CI)
Myocardial infarction (MI)		
Model 1 (non-drinker reference group)	Non-drinker (Ref.)	1.0 (Ref.)
	**Occasional**	**0.72 (0.54–0.96)**
	Light	1.04 (0.78–1.38)
	**Moderate**	**0.57 (0.33–0.98)**
	Heavy	0.95 (0.54–1.68)
Model 2 (abstainer reference group)	Abstainer: never (Ref.)	1.0 (Ref.)
	Former	0.89 (0.64–1.24)
	**Occasional**	**0.68 (0.48–0.96)**
	Light	0.96 (0.68–1.38)
	**Moderate**	**0.53 (0.29–0.95)**
	Heavy	0.88 (0.47–1.62)
Model 3 (Model 2 + occasional drinker reference group)	Former	1.32 (0.96–1.81)
	Occasional (Ref.)	1.0 (Ref.)
	**Light**	**1.43 (1.02–1.98)**
	Moderate	0.78 (0.44–1.37)
	Heavy	1.3 (0.72–2.33)
Model 4 (Model 3 + reallocation of former drinkers)	Occasional (Ref.)	1.0 (Ref.)
	**Light**	**1.46 (1.11–1.92)**
	Moderate	0.64 (0.38–1.06)
	Heavy	1.42 (0.95–2.13)
Stroke		
Model 1 (non-drinker reference group)	Non-drinker (Ref.)	1.0 (Ref.)
	Occasional	0.82 (0.62–1.1)
	Light	1.23 (0.91–1.65)
	Moderate	1.24 (0.79–1.96)
	Heavy	0.86 (0.41–1.79)
Model 2 (abstainer reference group)	Abstainer: never (Ref.)	1.0 (Ref.)
	Former	1.09 (0.78–1.51)
	Occasional	0.86 (0.61–1.22)
	Light	1.29 (0.90–1.85)
	Moderate	1.31 (0.79–2.17)
	Heavy	0.91 (0.42–1.96)
Model 3 (Model 2 + occasional drinker reference group)	Former	1.26 (0.92–1.74)
	Occasional (Ref.)	1.0 (Ref.)
	**Light**	**1.50 (1.08–2.1)**
	Moderate	1.52 (0.95–2.45)
	Heavy	1.06 (0.50–2.23)
Model 4 (Model 3 + reallocation of former drinkers)	Occasional (Ref.)	1.0 (Ref.)
	**Light**	**1.54 (1.17–2.03)**
	**Moderate**	**1.56 (1.05–2.33)**
	Heavy	0.90 (0.52–1.59)
Cardiovascular mortality		
Model 1 (non-drinker reference group)	Non-drinker (Ref.)	1.0 (Ref.)
	**Occasional**	**0.74 (0.57–0.96)**
	Light	0.86 (0.65–1.14)
	**Moderate**	**0.57 (0.34–0.97)**
	Heavy	0.86 (0.47–1.57)
Model 2 (abstainer reference group)	Abstainer: never (Ref.)	1.0 (Ref.)
	Former	1.17 (0.88–1.56)
	Occasional	0.81 (0.59–1.11)
	Light	0.95 (0.68–1.33)
	Moderate	0.64 (0.36–1.12)
	Heavy	0.96 (0.51–1.82)
Model 3 (Model 2 + occasional drinker reference group)	**Former**	**1.44 (1.09–1.91)**
	Occasional (Ref.)	1.0 (Ref.)
	Light	1.17 (0.85–1.61)
	Moderate	0.79 (0.46–1.35)
	Heavy	1.19 (0.64–2.2)
Model 4 (Model 3 + reallocation of former drinkers)	Occasional (Ref.)	1.0 (Ref.)
	Light	1.21 (0.94–1.56)
	Moderate	0.81 (0.53–1.25)
	Heavy	1.18 (0.78–1.76)
MACE (MI, stroke, CV mortality)		
Model 1 (non-drinker reference group)	Non-drinker (Ref.)	1.0 (Ref.)
	**Occasional**	**0.75 (0.63–0.9)**
	Light	1.00 (0.83–1.2)
	**Moderate**	**0.72 (0.52–0.99)**
	Heavy	0.90 (0.61–1.33)
Model 2 (abstainer reference group)	Abstainer: never (Ref.)	1.0 (Ref.)
	Former	1.06 (0.87–1.3)
	**Occasional**	**0.78 (0.63–0.96)**
	Light	1.04 (0.83–1.3)
	Moderate	0.75 (0.53–1.06)
	Heavy	0.94 (0.62–1.43)
Model 3 (Model 2 + occasional drinker reference group)	**Former**	**1.36 (1.13–1.65)**
	Occasional (Ref.)	1.0 (Ref.)
	**Light**	**1.33 (1.08–1.64)**
	Moderate	0.96 (0.69–1.33)
	Heavy	1.21 (0.81–1.8)
Model 4 (Model 3 + reallocation of former drinkers)	Occasional (Ref.)	1.0 (Ref.)
	**Light**	**1.33 (1.12–1.57)**
	Moderate	0.93 (0.71–1.23)
	Heavy	1.21 (0.92–1.6)
Heart failure		
Model 1 (non-drinker reference group)	Non-drinker (Ref.)	1.0 (Ref.)
	**Occasional**	**0.70 (0.53–0.91)**
	Light	0.87 (0.65–1.16)
	Moderate	1.03 (0.68–1.55)
	Heavy	1.15 (0.67–1.96)
Model 2 (abstainer reference group)	Abstainer: never (Ref.)	1.0 (Ref.)
	Former	1.35 (0.99–1.84)
	Occasional	0.84 (0.60–1.19)
	Light	1.06 (0.74–1.52)
	Moderate	1.27 (0.79–2.03)
	Heavy	1.43 (0.80–2.58)
Model 3 (Model 2 + occasional drinker reference group)	**Former**	**1.60 (1.2–2.14)**
	Occasional (Ref.)	1.0 (Ref.)
	Light	1.26 (0.91–1.76)
	Moderate	1.51 (0.97–2.34)
	Heavy	1.7 (0.98–2.97)
Model 4 (Model 3 + reallocation of former drinkers)	Occasional (Ref.)	1.0 (Ref.)
	**Light**	**1.42 (1.1–1.84)**
	Moderate	1.29 (0.88–1.88)
	**Heavy**	**1.63 (1.11–2.4)**

Hazard ratios (HR) and 95% confidence intervals (CIs) for incident cardiovascular outcomes, including myocardial infarction (MI), stroke, cardiovascular mortality, major adverse cardiovascular events (MACEs), and heart failure. Risk estimates are compared across four models progressively addressing abstainer and sick-quitter biases. Bolded cells refer to statistically significant associations at a two-sided α = 0.05 threshold.

## Data Availability

The data used in this study cannot be publicly shared; however, it will be shared to investigators upon request through the multi-ethnic study of atherosclerosis website: https://www.mesa-nhlbi.org/.

## References

[1] Esser MB, Leung G, Sherk A, Bohm MK, Liu Y, Lu H, Estimated deaths attributable to excessive alcohol use among US adults aged 20 to 64 years, 2015 to 2019. JAMA Netw Open 2022;5:e2239485. 10.1001/jamanetworkopen.2022.39485 PMC9627409 36318209

[2] Marti-Aguado D, Calleja JL, Vilar-Gomez E, Iruzubieta P, Rodríguez-Duque JC, Del Barrio M, Low-to-moderate alcohol consumption is associated with increased fibrosis in individuals with metabolic dysfunction-associated steatotic liver disease. J Hepatol 2024;81:930–940. 10.1016/j.jhep.2024.06.036 38971533

[3] Cecchini M, Filippini T, Whelton PK, Iamandii I, Di Federico S, Boriani G, Alcohol intake and risk of hypertension: a systematic review and dose-response meta-analysis of nonexperimental cohort studies. Hypertens Dallas Tex 1979 2024;81:1701–1715. 10.1161/HYPERTENSIONAHA.124.22703 PMC11251509 38864208

[4] O’Keefe JH, Bhatti SK, Bajwa A, DiNicolantonio JJ, Lavie CJ. Alcohol and cardiovascular health: the dose makes the poison…or the remedy. Mayo Clin Proc 2014;89:382–393. 10.1016/j.mayocp.2013.11.005 24582196

[5] Ronksley PE, Brien SE, Turner BJ, Mukamal KJ, Ghali WA. Association of alcohol consumption with selected cardiovascular disease outcomes: a systematic review and meta-analysis. The BMJ 2011;342:d671. 10.1136/bmj.d671 PMC3043109 21343207

[6] Carr JJ, Nelson JC, Wong ND, McNitt-Gray M, Arad Y, Jacobs DR, Calcified coronary artery plaque measurement with cardiac CT in population-based studies: standardized protocol of Multi-Ethnic Study of Atherosclerosis (MESA) and Coronary Artery Risk Development in Young Adults (CARDIA) study. Radiology 2005;234:35–43. 10.1148/radiol.2341040439 15618373

[7] Djoussé L, Gaziano JM. Alcohol consumption and heart failure. Curr Atheroscler Rep 2008;10:117–120. 10.1007/s11883-008-0017-z PMC2365733 18417065

[8] Carr S, Bryazka D, McLaughlin SA, Zheng P, Bahadursingh S, Aravkin AY, A burden of proof study on alcohol consumption and ischemic heart disease. Nat Commun 2024;15:4082. 10.1038/s41467-024-47632-7 PMC11094064 38744810

[9] van de Luitgaarden IAT, van Oort S, Bouman EJ, Bouman EJ, Schoonmade LJ, Schrieks IC, Alcohol consumption in relation to cardiovascular diseases and mortality: a systematic review of Mendelian randomization studies. Eur J Epidemiol 2022;37:655–669. 10.1007/s10654-021-00799-5 PMC9329419 34420153

[10] Biddinger KJ, Emdin CA, Haas ME, Wang M, Hindy G, Ellinor PT, Association of habitual alcohol intake with risk of cardiovascular disease. JAMA Netw Open 2022;5:e223849. 10.1001/jamanetworkopen.2022.3849 PMC8956974 35333364

[11] Naimi TS, Brown DW, Brewer RD, Giles WH, Mensah G, Serdula MK, Cardiovascular risk factors and confounders among nondrinking and moderate-drinking U.S. adults. Am J Prev Med 2005;28:369–373. 10.1016/j.amepre.2005.01.011 15831343

[12] Ng Fat L, Cable N, Shelton N. Worsening of health and a cessation or reduction in alcohol consumption to special occasion drinking across three decades of the life course. Alcohol Clin Exp Res 2015;39:166–174. 10.1111/acer.12596 PMC4329335 25623415

[13] Fan AZ, Ruan WJ, Chou SP. Re-examining the relationship between alcohol consumption and coronary heart disease with a new lens. Prev Med 2019; 118:336–343. 10.1016/j.ypmed.2018.11.022 PMC7571539 30508551

[14] Naimi TS, Chikritzhs T. Santa Claus, the Tooth Fairy, and purported lifetime nondrinkers: ramifications for observational evidence about alcohol and health. Alcohol Clin Exp Res 2025;49:92–94. 10.1111/acer.15478 39500830

[15] Sarich P, Gao S, Zhu Y, Canfell K, Weber MF. The association between alcohol consumption and all-cause mortality: an umbrella review of systematic reviews using lifetime abstainers or low-volume drinkers as a reference group. Addict Abingdon Engl 2024;119:998–1012. 10.1111/add.16446 38465993

[16] Rehm J, Irving H, Ye Y, Kerr WC, Bond J, Greenfield TK. Are lifetime abstainers the best control group in alcohol epidemiology? On the stability and validity of reported lifetime abstention. Am J Epidemiol 2008;168:866–871. 10.1093/aje/kwn093 PMC2565735 18701442

[17] Xi B, Veeranki SP, Zhao M, Ma C, Yan Y, Mi J. Relationship of alcohol consumption to all-cause, cardiovascular, and cancer-related mortality in U.S. adults. J Am Coll Cardiol 2017;70:913–922. 10.1016/j.jacc.2017.06.054 28818200

[18] Mukamal KJ, Chen CM, Rao SR, Breslow RA. Alcohol consumption and cardiovascular mortality among U.S. adults, 1987–2002. J Am Coll Cardiol 2010;55: 1328–1335. 10.1016/j.jacc.2009.10.056 PMC3865979 20338493

[19] McClelland RL, Bild DE, Burke GL, Mukamal KJ, Lima JA, Kronmal RA. Alcohol and coronary artery calcium prevalence, incidence and progression: results from the Multi-Ethnic Study of Atherosclerosis (MESA). Am J Clin Nutr 2008;88:1593–1601. 10.3945/ajcn.2008.26420 PMC3319440 19064520

[20] Kerr WC, Lui CK, Williams E, Ye Y, Greenfield TK, Lown EA. Health risk factors associated with lifetime abstinence from alcohol in the 1979 National Longitudinal Survey of Youth Cohort. Alcohol Clin Exp Res 2017;41:388–398. 10.1111/acer.13302 PMC5272800 28063241

[21] Fillmore KM, Kerr WC, Bostrom A. Changes in drinking status, serious illness and mortality. J Stud Alcohol 2003;64:278–285. 10.15288/jsa.2003.64.278 12713203

[22] Shaper AG. Alcohol consumption decreases with the development of disease. Addiction 2011;106:1023–1025. 10.1111/j.1360-0443.2011.03372.x 21382114

[23] Shaper AG, Wannamethee G, Walker M. Alcohol and mortality in British men: explaining the U-shaped curve. Lancet Lond Engl 1988;2:1267–1273. 10.1016/s0140-6736(88)92890-5 2904004

[24] Caldwell TM, Rodgers B, Power C, Clark C, Stansfeld SA. Drinking histories of self-identified lifetime abstainers and occasional drinkers: findings from the 1958 British birth cohort study. Alcohol Alcohol Oxf Oxfs 2006;41:650–654. 10.1093/alcalc/agl088 17028305

[25] Callinan S, Chikritzhs T, Livingston M. Consistency of drinker Status over time: drinking patterns of ex-drinkers who describe themselves as lifetime abstainers. J Stud Alcohol Drugs 2019;80:552–556. 31603757

[26] Ekholm O, Bloomfield K, Thygesen LC. Alcohol habits and alcohol-related health conditions of self-defined lifetime abstainers and never binge drinkers. Alcohol Clin Exp Res 2024;48:1905–1914. 10.1111/acer.15433 39231784

[27] Naimi T, Chikritzhs T, Stockwell T. Commentary on Di Castelnuovo : implications of using low volume drinkers instead of never drinkers as the reference group. Addiction 2022;117:327–329. 10.1111/add.15692 34658091

[28] Liang W, Chikritzhs T. Observational research on alcohol use and chronic disease outcome: new approaches to counter biases. Sci World J 2013;2013: 860915. 10.1155/2013/860915 PMC3725844 23935438

[29] Park JE, Ryu Y, Cho SI. The effect of reference group classification and change in alcohol consumption on the association between alcohol consumption and cardiovascular disease. Alcohol Clin Exp Res 2017;41:379–387. 10.1111/acer.13299 28098956

[30] Shaper AG, Wannamethee SG. Alcohol intake and mortality in middle aged men with diagnosed coronary heart disease. Heart Br Card Soc 2000;83: 394–399. 10.1136/heart.83.4.394 PMC1729361 10722536

[31] Goulden R. Moderate alcohol consumption is not associated with reduced all-cause mortality. Am J Med 2016;129:180–186.e4. 10.1016/j.amjmed.2015.10.013 26524703

[32] Ortolá R, Sotos-Prieto M, García-Esquinas E, Galán I, Rodríguez-Artalejo F. Alcohol consumption patterns and mortality among older adults with health-related or socioeconomic risk factors. JAMA Netw Open 2024;7: e2424495. 10.1001/jamanetworkopen.2024.24495 PMC11320169 39133491

[33] Clay JM, Callaghan RC, Sherk A, Naimi TS, Stockwell T, Asbridge M. Alcohol consumption and mortality among Canadian drinkers: a national population-based survival analysis (2000–2017). Drug Alcohol Rev 2025;44:434–447. 10.1111/dar.13993 PMC11814365 39667732

[34] Naimi TS, Stockwell T, Zhao J, Xuan Z, Dangardt F, Saitz R, Selection biases in observational studies affect associations between “moderate” alcohol consumption and mortality. Addict Abingdon Engl 2017;112:207–214. 10.1111/add.13451 27316346

[35] Di Castelnuovo A, Costanzo S, Bonaccio M, McElduff P, Linneberg A, Salomaa V, Alcohol intake and total mortality in 142 960 individuals from the MORGAM project: a population-based study. Addict Abingdon Engl 2022; 117:312–325. 10.1111/add.15593 34105209

[36] Kerr WC, Ye Y. Relationship of life-course drinking patterns to diabetes, heart problems, and hypertension among those 40 and older in the 2005 U.S. National Alcohol Survey. J Stud Alcohol Drugs 2010;71:515–525. 10.15288/jsad.2010.71.515 PMC2887921 20553659

[37] Bild DE, Bluemke DA, Burke GL, Detrano R, Diez Roux AV, Folsom AR, Multi-ethnic study of atherosclerosis: objectives and design. Am J Epidemiol 2002;156:871–881. 10.1093/aje/kwf113 12397006

[38] Budoff MJ, Kinninger A, Gransar H, Achenbach S, Al-Mallah M, Bax JJ, When does a calcium score equate to secondary prevention?: insights from the multinational CONFIRM registry. JACC Cardiovasc Imaging 2023;16: 1181–1189. 10.1016/j.jcmg.2023.03.008 37227328

[39] Gaine SP, Blumenthal RS, Sharma G. Coronary artery calcium score as a graded decision tool. JACC Adv 2023;2:100664. 10.1016/j.jacadv.2023.100664 PMC11198403 38938721

[40] Detrano R, Guerci AD, Carr JJ, Bild DE, Burke G, Folsom AR, Coronary calcium as a predictor of coronary events in four racial or ethnic groups. N Engl J Med 2008;358:1336–1345. 10.1056/NEJMoa072100 18367736

[41] Kerr WC, Fillmore KM, Bostrom A. Stability of alcohol consumption over time: evidence from three longitudinal surveys from the United States. J Stud Alcohol 2002;63:325–333. 10.15288/jsa.2002.63.325 12086133

[42] Voskoboinik A, Kalman JM, Silva AD, Nicholls T, Costello B, Nanayakkara S, Alcohol abstinence in drinkers with atrial fibrillation. N Engl J Med 2020;382:20–28. 10.1056/NEJMoa1817591 31893513

