# Supplementary material for: Reassessing alcohol consumption and cardiovascular disease by addressing bias in observational data: results from the multi-ethnic study of atherosclerosis
Source: Eur J Prev Cardiol. Author manuscript; Available in PMC 2026 Jul 28. (PMC13409341; doi:10.1093/eurjpc/zwag201)
Supplement: supplementary data [file NIHMS2197981-supplement-supplementary_data.docx]

**Supplemental Table 1. Comparison between Moderate Drinkers and Abstainer vs Occasional Reference Groups**

|  | | Abstainer  (N=1390) | Current-Occasional  ( <=1 drink/wk)  (N=1781) | Current-Moderate  (8 – 14 drinks/wk)  (N=411) | p-value  (Abstainer vs. Moderate) | p-value  (Occasional vs. Moderate) | p-value  (Abstainer vs. Occasional) |
| --- | --- | --- | --- | --- | --- | --- | --- |
| Age at Enrollment, Mean (SD) | | 63.9 ± 10.2 | 60.8 ± 10.2 | 62.6 ± 10.1 | **0.001** | **0.02** | **<0.001** |
| Sex, n (%) | *Female* | 1065 (76.6%) | 1029 (57.8%) | 206 (34.5%) | **<0.001** | **<0.001** | **<0.001** |
|  | *Male* | 325 (23.4%) | 752 (42.2%) | 391 (65.5%) |  |  |  |
| Race/Ethnicity, n (%) | *White* | 246 (17.7%) | 750 (42.1%) | 357 (59.8%) | **<0.001** | **<0.001** | **<0.001** |
|  | *Chinese* | 432 (31.1%) | 177 (9.9%) | 22 (3.7%) |  |  |  |
|  | *Black* | 324 (23.3%) | 481 (27.0%) | 126 (21.1%) |  |  |  |
|  | *Hispanic/Latino* | 388 (27.9%) | 373 (20.9%) | 92 (15.4%) |  |  |  |
| Education,  n (%) | *>= Bachelor’s Degree* | 321 (23.1%) | 706 (39.6%) | 193 (47.0%) | **<0.001** | **0.007** | **<0.001** |
| Income,  n (%) | >= $75,000 | 128 (9.6%) | 429 (24.8%) | 158 (39.3%) | **<0.001** | **<0.001** | **<0.001** |
| Body Mass Index (kg)/(m^2), Mean (SD) | | 27.9 ± 5.7 | 28.6 ± 5.6 | 27.5 ± 4.8 | 0.774 | **0.002** | **<0.001** |
| Seated Systolic Blood Pressure (mmHg), Mean (SD) | | 129.6 ± 22.9 | 124.4 ± 20.6 | 125.2 ± 21.2 | **0.001** | 0.463 | **<0.001** |
| Seated Diastolic Blood Pressure (mmHg),  Mean (SD) | | 70.9 ± 10.3 | 71.2 ± 9.9 | 73.6 ± 10.4 | **<0.001** | **<0.001** | 0.284 |
| Blood Pressure Medication Use,  n (%) | | 519 (37.3%) | 535 (30.0%) | 129 (31.4%) | **0.035** | 0.577 | **<0.001** |
| Diabetes,  n (%) | | 197 (14.2%) | 165 (9.3%) | 25 (6.1%) | **<0.001** | 0.079 | **<0.001** |
| Cancer,  n (%) | | 87 (6.3%) | 124 (7.0%) | 39 (9.5%) | **0.056** | 0.192 | 0.724 |
| Liver disease,  n (%) | | 51 (3.7%) | 44 (2.5%) | 16 (3.9%) | **0.648** | 0.214 | **0.017** |
| Smoking (Pack-Years), Mean (SD) | | 3.7 ± 13.2 | 4.0 ± 5.9 | 3.1 ± 4.3 | **<0.001** | **<0.001** | **<0.001** |
| Lipid-Lowering Medication Use,  n (%) | | 223 (16.0%) | 298 (16.8%) | 63 (15.3%) | 0.754 | 0.514 | 0.589 |
| LDL Cholesterol (mg/dL),  Mean (SD) | | 118.0 ± 32.0 | 118.1 ± 31.4 | 115.8 ± 31.6 | 0.388 | 0.262 | 0.765 |
| C-Reactive Protein (CRP) (mg/L),  Mean (SD) | | 3.7 ± 5.0 | 4.0 ± 5.9 | 3.1 ± 4.3 | **0.007** | **0.001** | 0.334 |
| Fibrinogen (mg/dL),  Mean (SD) | | 360.5 ± 71.7 | 347.1 ± 71.9 | 321.7 ± 69.3 | **<0.001** | **<0.001** | **<0.001** |

**Supplemental Figure 1: Sensitivity Analysis for CAC Score Cutoffs (0, 1-10, 11-100, >100)**

**
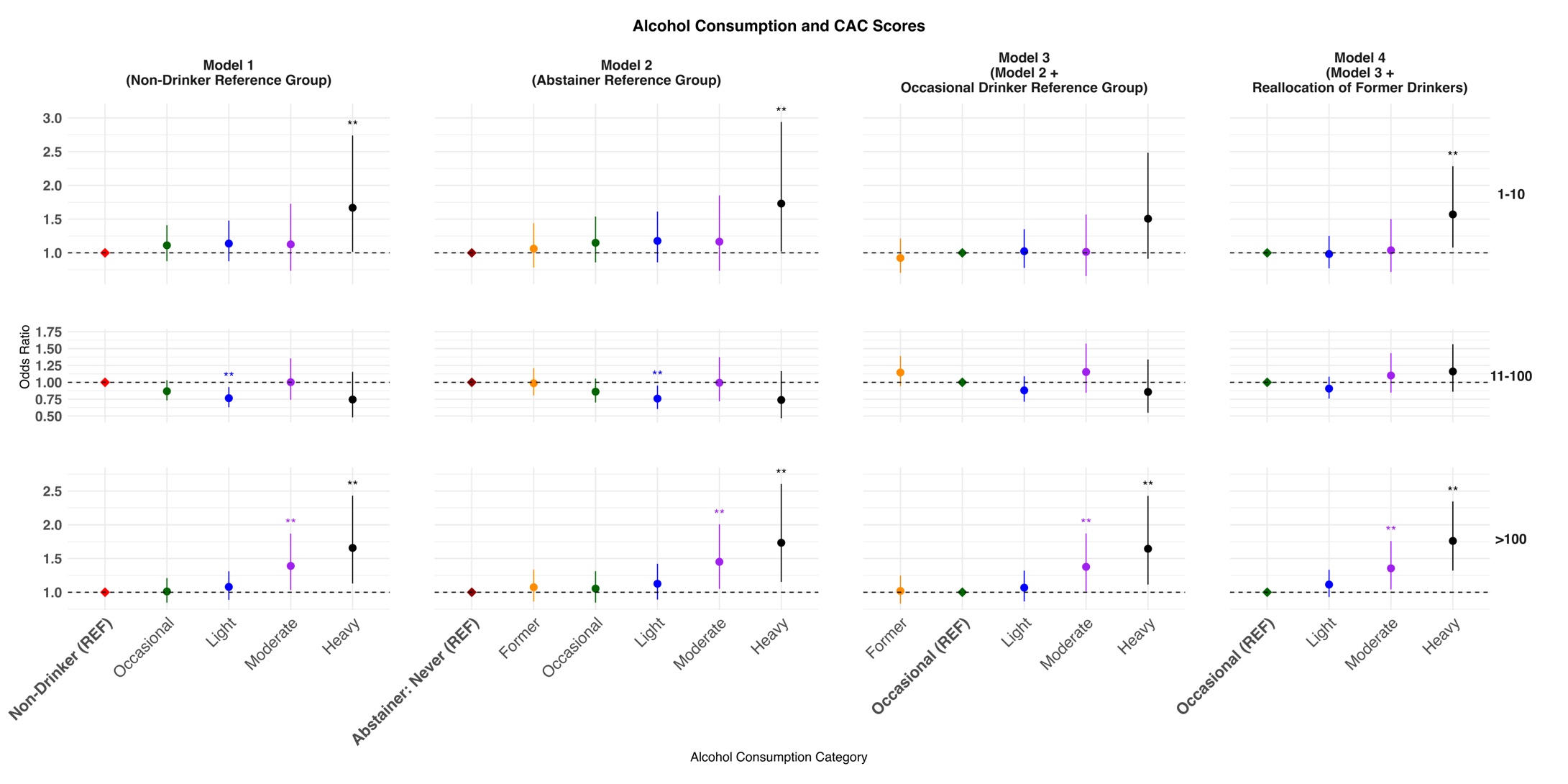
**

**Supplemental Table 2: Sensitivity Analysis for CAC Score Cutoffs (0, 1-10, 11-100, >100)**

|  | Drinking Level | OR (95% CI) |
| --- | --- | --- |
| **1-10** | | |
| **Model 1 (Non-Drinker Reference Group)** | **Non-Drinker (REF)** | OR: 1.0 (Reference) |
|  | Occasional | OR: 1.11 (0.88-1.41) |
|  | Light | OR: 1.14 (0.88-1.48) |
|  | Moderate | OR: 1.13 (0.73-1.73) |
|  | Heavy | **OR: 1.67 (1.02-2.74)** |
| **Model 2 (Abstainer Reference Group)** | **Abstainer: Never (REF)** | OR: 1.0 (Reference) |
|  | Former | OR: 1.06 (0.78-1.44) |
|  | Occasional | OR: 1.15 (0.86-1.54) |
|  | Light | OR: 1.18 (0.86-1.61) |
|  | Moderate | OR: 1.17 (0.73-1.85) |
|  | Heavy | **OR: 1.73 (1.02-2.94)** |
| **Model 3 (Model 2 + Occasional Drinker Reference Group)** | Former | OR: 0.92 (0.7-1.21) |
|  | **Occasional (REF)** | OR: 1.0 (Reference) |
|  | Light | OR: 1.02 (0.78-1.35) |
|  | Moderate | OR: 1.01 (0.66-1.57) |
|  | Heavy | OR: 1.51 (0.91-2.48) |
| **Model 4 (Model 3 + Reallocation of Former Drinkers)** | **Occasional (REF)** | OR: 1.0 (Reference) |
|  | Light | OR: 0.98 (0.77-1.25) |
|  | Moderate | OR: 1.04 (0.72-1.5) |
|  | Heavy | **OR: 1.57 (1.08-2.28)** |
| **11-100** | | |
| **Model 1 (Non-Drinker Reference Group)** | **Non-Drinker (REF)** | OR: 1.0 (Reference) |
|  | Occasional | OR: 0.87 (0.73-1.03) |
|  | Light | **OR: 0.77 (0.63-0.93)** |
|  | Moderate | OR: 1 (0.74-1.35) |
|  | Heavy | OR: 0.75 (0.48-1.16) |
| **Model 2 (Abstainer Reference Group)** | **Abstainer: Never (REF)** | OR: 1.0 (Reference) |
|  | Former | OR: 0.99 (0.81-1.21) |
|  | Occasional | OR: 0.86 (0.7-1.06) |
|  | Light | **OR: 0.76 (0.61-0.95)** |
|  | Moderate | OR: 0.99 (0.72-1.37) |
|  | Heavy | OR: 0.74 (0.47-1.17) |
| **Model 3 (Model 2 + Occasional Drinker Reference Group)** | Former | OR: 1.15 (0.94-1.39) |
| **Model 3 (Model 2 + Occasional Drinker Reference Group)** | **Occasional (REF)** | OR: 1.0 (Reference) |
|  | Light | OR: 0.88 (0.71-1.09) |
|  | Moderate | OR: 1.15 (0.84-1.58) |
|  | Heavy | OR: 0.86 (0.55-1.34) |
| **Model 4 (Model 3 + Reallocation of Former Drinkers)** | **Occasional (REF)** | OR: 1.0 (Reference) |
|  | Light | OR: 0.91 (0.76-1.08) |
|  | Moderate | OR: 1.1 (0.85-1.43) |
|  | Heavy | OR: 1.16 (0.86-1.57) |
| **>100** | | |
| **Model 1 (Non-Drinker Reference Group)** | **Non-Drinker (REF)** | OR: 1.0 (Reference) |
|  | Occasional | OR: 1.01 (0.85-1.21) |
|  | Light | OR: 1.08 (0.89-1.31) |
|  | Moderate | **OR: 1.39 (1.03-1.87)** |
|  | Heavy | **OR: 1.66 (1.13-2.43)** |
| **Model 2 (Abstainer Reference Group)** | **Abstainer: Never (REF)** | OR: 1.0 (Reference) |
|  | Former | OR: 1.07 (0.86-1.34) |
|  | Occasional | OR: 1.05 (0.85-1.31) |
|  | Light | OR: 1.13 (0.89-1.42) |
|  | Moderate | **OR: 1.45 (1.05-2.01)** |
|  | Heavy | **OR: 1.73 (1.15-2.6)** |
| **Model 3 (Model 2 + Occasional Drinker Reference Group)** | Former | OR: 1.02 (0.83-1.25) |
|  | **Occasional (REF)** | OR: 1.0 (Reference) |
|  | Light | OR: 1.07 (0.86-1.32) |
|  | Moderate | **OR: 1.38 (1.01-1.87)** |
|  | Heavy | **OR: 1.64 (1.11-2.43)** |
| **Model 4 (Model 3 + Reallocation of Former Drinkers)** | **Occasional (REF)** | OR: 1.0 (Reference) |
|  | Light | OR: 1.11 (0.93-1.33) |
|  | Moderate | **OR: 1.35 (1.04-1.76)** |
|  | Heavy | **OR: 1.76 (1.32-2.35)** |

**Supplemental Figure 2: Fine Gray Sub-Distribution Hazard Ratios for Alcohol Consumption and Incident Cardiovascular outcomes**

**
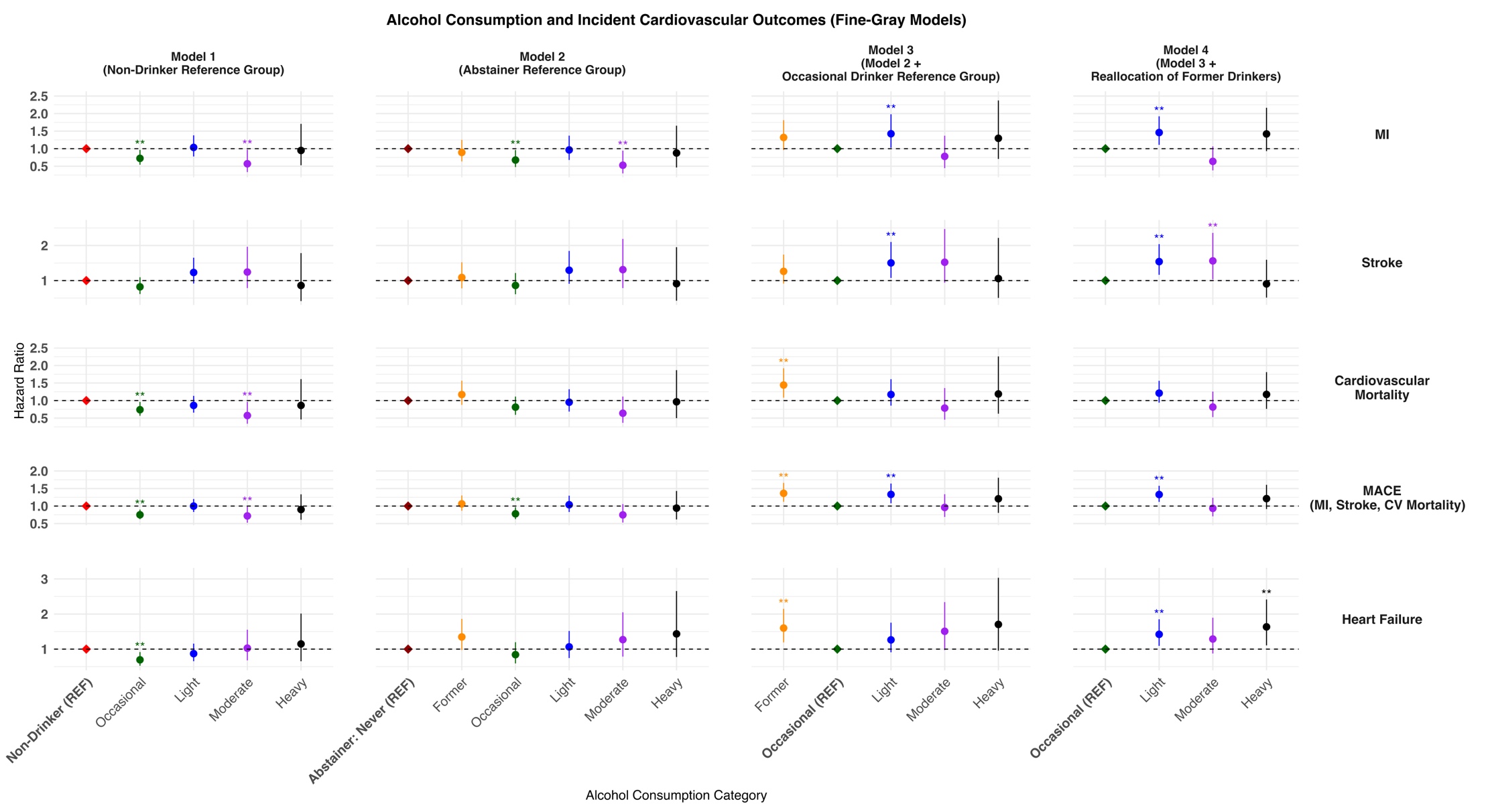
**

**Supplemental Table 3: Fine Gray Sub-distribution Hazard Ratios**

| Model | Drinking Level | HR (95% CI) |
| --- | --- | --- |
| **MI** | | |
| **Model 1** | **Non-Drinker (REF)** | HR: 1 (Reference) |
|  | **Occasional** | **HR: 0.72 (0.54-0.96)** |
|  | Light | HR: 1.04 (0.78-1.38) |
|  | **Moderate** | **HR: 0.57 (0.33-0.97)** |
|  | Heavy | HR: 0.95 (0.53-1.71) |
| **Model 2** | **Abstainer: Never (REF)** | HR: 1 (Reference) |
|  | Former | HR: 0.89 (0.64-1.25) |
|  | **Occasional** | **HR: 0.68 (0.48-0.96)** |
|  | Light | HR: 0.96 (0.68-1.37) |
|  | **Moderate** | **HR: 0.53 (0.3-0.94)** |
|  | Heavy | HR: 0.88 (0.46-1.66) |
| **Model 3** | Former | HR: 1.32 (0.96-1.81) |
|  | **Occasional (REF)** | HR: 1 (Reference) |
|  | **Light** | **HR: 1.43 (1.03-1.98)** |
|  | Moderate | HR: 0.78 (0.44-1.37) |
|  | Heavy | HR: 1.3 (0.71-2.37) |
| **Model 4** | **Occasional (REF)** | HR: 1 (Reference) |
|  | **Light** | **HR: 1.46 (1.11-1.92)** |
|  | Moderate | HR: 0.64 (0.38-1.07) |
|  | Heavy | HR: 1.42 (0.93-2.17) |
| **Stroke** | | |
| **Model 1** | **Non-Drinker (REF)** | HR: 1 (Reference) |
|  | Occasional | HR: 0.82 (0.62-1.09) |
|  | Light | HR: 1.23 (0.92-1.65) |
|  | Moderate | HR: 1.24 (0.79-1.96) |
|  | Heavy | HR: 0.86 (0.42-1.78) |
| **Model 2** | **Abstainer: Never (REF)** | HR: 1 (Reference) |
|  | Former | HR: 1.09 (0.78-1.52) |
|  | Occasional | HR: 0.86 (0.61-1.21) |
|  | Light | HR: 1.29 (0.91-1.84) |
|  | Moderate | HR: 1.31 (0.79-2.19) |
|  | Heavy | HR: 0.91 (0.42-1.95) |
| **Model 3** | Former | HR: 1.26 (0.92-1.74) |
|  | **Occasional (REF)** | HR: 1 (Reference) |
|  | **Light** | **HR: 1.5 (1.08-2.1)** |
|  | Moderate | HR: 1.52 (0.94-2.47) |
|  | Heavy | HR: 1.06 (0.5-2.22) |
| **Model 4** | **Occasional (REF)** | HR: 1 (Reference) |
|  | **Light** | **HR: 1.54 (1.16-2.04)** |
|  | **Moderate** | **HR: 1.56 (1.03-2.36)** |
|  | Heavy | HR: 0.9 (0.51-1.59) |
| **Cardiovascular Mortality** | | |
| **Model 1** | **Non-Drinker (REF)** | HR: 1 (Reference) |
|  | **Occasional** | **HR: 0.74 (0.57-0.96)** |
|  | Light | HR: 0.86 (0.65-1.13) |
|  | **Moderate** | **HR: 0.57 (0.34-0.97)** |
|  | Heavy | HR: 0.86 (0.46-1.61) |
| **Model 2** | **Abstainer: Never (REF)** | HR: 1 (Reference) |
|  | Former | HR: 1.17 (0.88-1.56) |
|  | Occasional | HR: 0.81 (0.59-1.11) |
|  | Light | HR: 0.95 (0.69-1.32) |
|  | Moderate | HR: 0.64 (0.37-1.11) |
|  | Heavy | HR: 0.96 (0.5-1.87) |
| **Model 3** | **Former** | **HR: 1.44 (1.08-1.92)** |
|  | **Occasional (REF)** | HR: 1 (Reference) |
|  | Light | HR: 1.17 (0.85-1.61) |
|  | Moderate | HR: 0.79 (0.46-1.36) |
|  | Heavy | HR: 1.19 (0.62-2.26) |
| **Model 4** | **Occasional (REF)** | HR: 1 (Reference) |
|  | Light | HR: 1.21 (0.94-1.56) |
|  | Moderate | HR: 0.81 (0.53-1.26) |
|  | Heavy | HR: 1.18 (0.76-1.81) |
| **MACE (MI, Stroke, CV Mortality)** | | |
| **Model 1** | **Non-Drinker (REF)** | HR: 1 (Reference) |
|  | Occasional | HR: 0.75 (0.63-0.9) |
|  | Light | HR: 1 (0.83-1.2) |
|  | Moderate | HR: 0.72 (0.52-0.98) |
|  | Heavy | HR: 0.9 (0.61-1.33) |
| **Model 2** | **Abstainer: Never (REF)** | HR: 1 (Reference) |
| **Model 2** | Former | HR: 1.06 (0.87-1.3) |
|  | Occasional | HR: 0.78 (0.63-0.97) |
|  | Light | HR: 1.04 (0.83-1.3) |
|  | Moderate | HR: 0.75 (0.53-1.06) |
|  | Heavy | HR: 0.94 (0.62-1.43) |
| **Model 3** | **Former** | **HR: 1.36 (1.12-1.66)** |
|  | **Occasional (REF)** | HR: 1 (Reference) |
|  | **Light** | **HR: 1.33 (1.08-1.64)** |
|  | Moderate | HR: 0.96 (0.69-1.34) |
|  | Heavy | HR: 1.21 (0.81-1.81) |
| **Model 4** | **Occasional (REF)** | HR: 1 (Reference) |
|  | **Light** | **HR: 1.33 (1.12-1.58)** |
|  | Moderate | HR: 0.93 (0.71-1.24) |
|  | Heavy | HR: 1.21 (0.91-1.61) |
| **Heart Failure** | | |
| **Model 1** | **Non-Drinker (REF)** | HR: 1 (Reference) |
|  | Occasional | HR: 0.70 (0.53-0.92) |
|  | Light | HR: 0.87 (0.66-1.16) |
|  | Moderate | HR: 1.03 (0.68-1.55) |
|  | Heavy | HR: 1.15 (0.65-2.01) |
| **Model 2** | **Abstainer: Never (REF)** | HR: 1 (Reference) |
|  | Former | HR: 1.35 (0.97-1.86) |
|  | Occasional | HR: 0.84 (0.59-1.20) |
|  | Light | HR: 1.06 (0.75-1.52) |
|  | Moderate | HR: 1.27 (0.79-2.05) |
|  | Heavy | HR: 1.43 (0.77-2.66) |
| **Model 3** | **Former** | **HR: 1.60 (1.19-2.15)** |
|  | **Occasional (REF)** | HR: 1 (Reference) |
|  | Light | HR: 1.26 (0.91-1.75) |
|  | Moderate | HR: 1.51 (0.97-2.34) |
|  | Heavy | HR: 1.70 (0.96-3.03) |
| **Model 4** | **Occasional (REF)** | HR: 1 (Reference) |
|  | **Light** | **HR: 1.42 (1.09-1.85)** |
|  | Moderate | HR: 1.29 (0.88-1.89) |
|  | **Heavy** | **HR: 1.63 (1.11-2.41)** |

**Supplemental Figure 3: Fine Gray Cumulative Incidence Curves for Alcohol Consumption and Incident Cardiovascular outcomes**

**
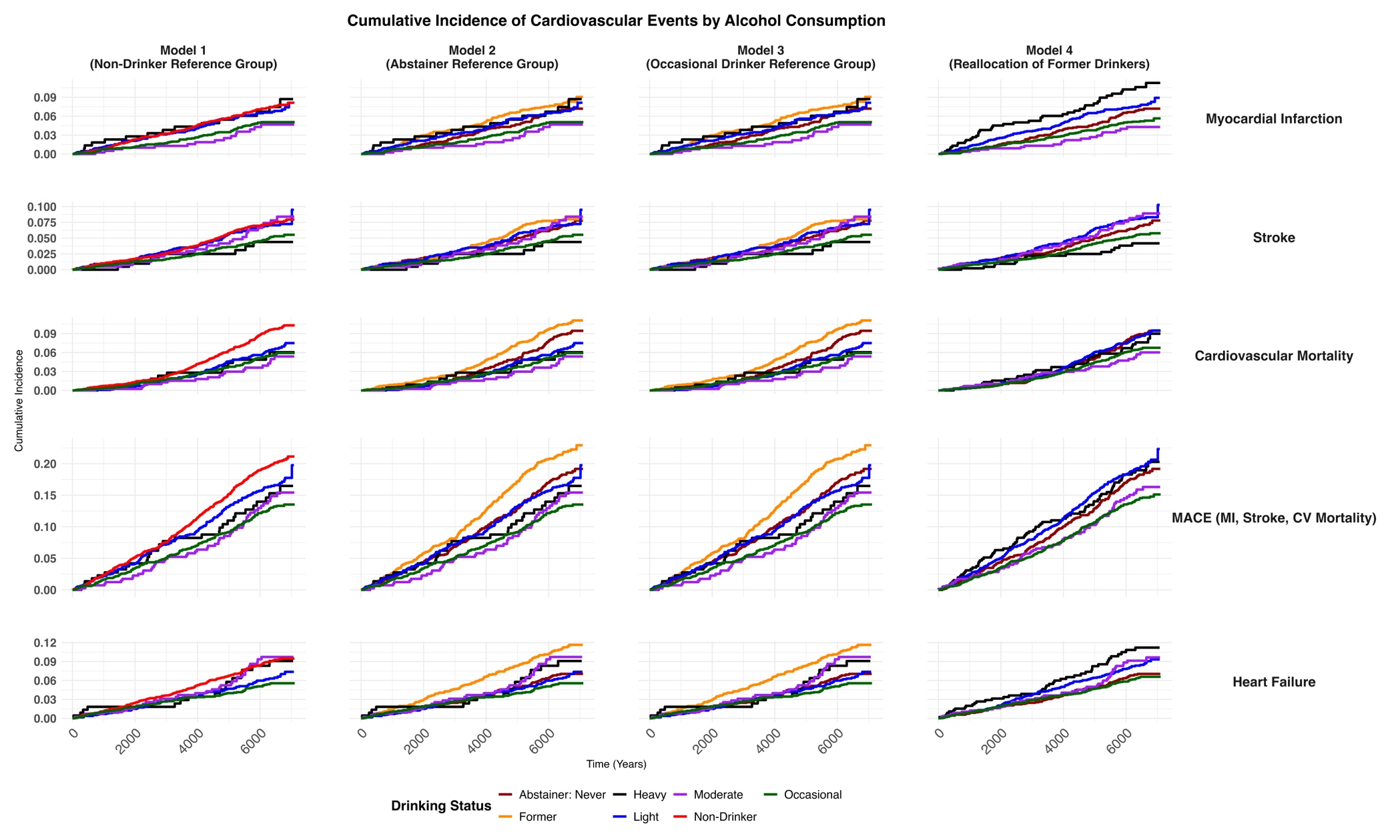
**

**Supplemental Figure 4. Gender Stratified Alcohol Consumption vs CAC Score**

**Supplemental Figure 5: Alcohol Consumption and Incident Cardiovascular outcomes stratified by Gender**

**
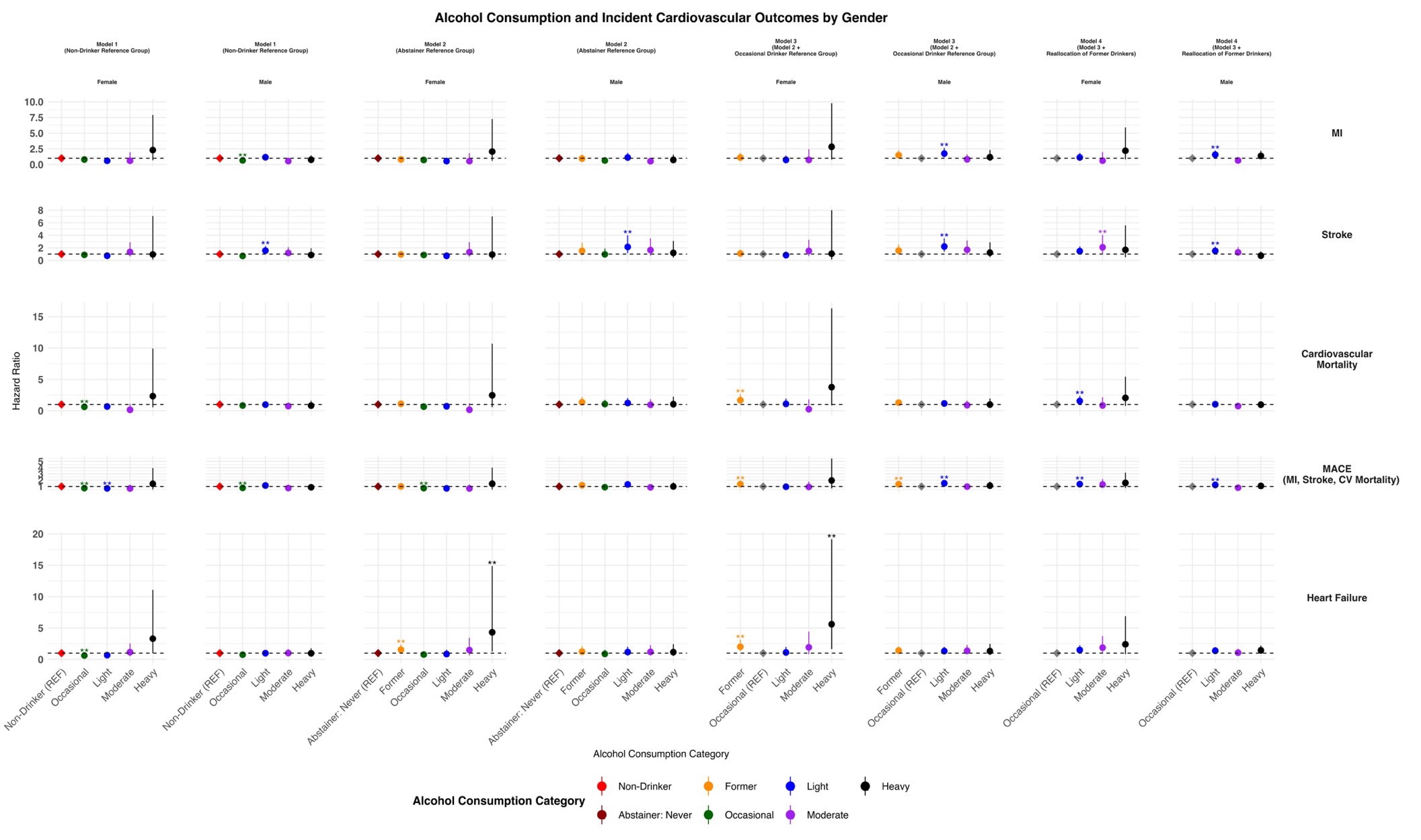
**

**Supplemental Figure 6: Alcohol Consumption and Incident Cardiovascular outcomes (Sensitivity analysis excluding abstainers who transitioned in status longitudinally)**

**
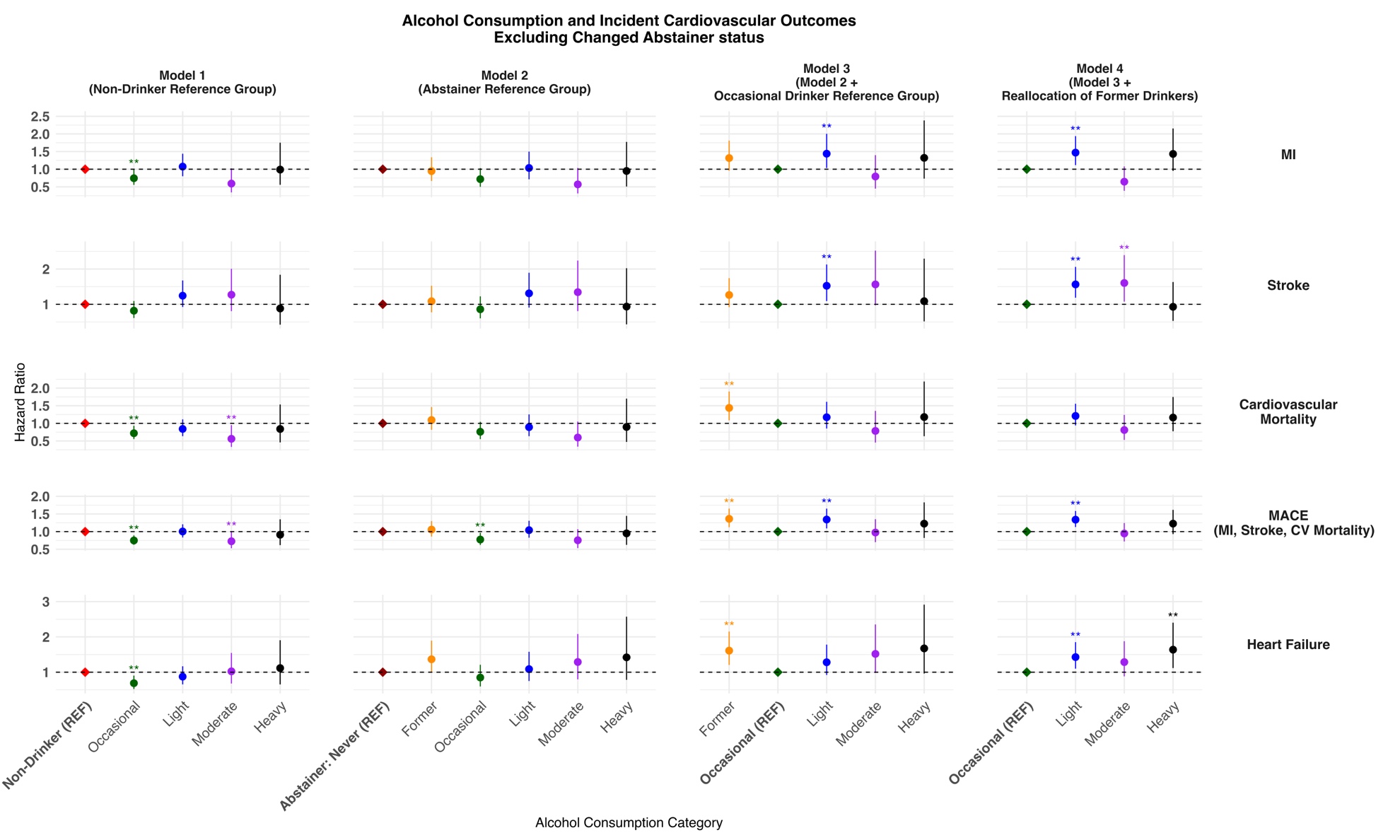
**
